# An Intracellular Peptide Library Screening Platform Identifies Irreversible Covalent Transcription Factor Inhibitors

**DOI:** 10.1002/advs.202416963

**Published:** 2025-03-17

**Authors:** Andrew Brennan, Scott Lovell, Keith W Vance, Jody M Mason

**Affiliations:** ^1^ Department of Life Sciences University of Bath Bath BA2 7AY UK

**Keywords:** activator protein‐1, cJun, covalent inhibitor, peptide antagonist, protein–protein interactions, transcription factor

## Abstract

The development of an intracellular peptide library screening platform is described to identify covalent transcription factor (TF) antagonists. The *Transcription Block Survival* (TBS) assay and subsequent hit refinement previously produced potent but reversible antagonists of the oncogenic TF cJun. TBS moves beyond a target binding readout to ensure loss of TF function by blocking TF‐DNA binding. Here, the TBS methodology is significantly expanded to identify covalent and highly selective inhibitors. A 131,072‐member library is probed containing a Cys option at nine positions within a non‐reducing cell line. This identified a single Cys residue with the appropriate geometry for disulphide bond formation with cJun C269 in its DNA binding domain. The selection of a unique Cys in the antagonist indicates both target shutdown and concomitant disulphide formation in a single step, resulting in increased potency. Substituting Cys with an electrophile generates an irreversible yet highly selective covalent cJun inhibitor capable of penetrating human melanoma cells in culture and depleting oncogenic cJun levels to inhibit cell viability, with enhanced efficacy compared to a previous cJun‐targeting peptide. This enhanced covalent‐TBS screening pipeline provides a robust approach to profile target protein surfaces for ligandable cysteines, producing covalent and selective antagonists with appropriately positioned warheads.

## Graphical abstract

A peptide library screening platform has been developed to discover covalent transcription factor inhibitors. It identifies an antagonist cysteine residue, which forms an inhibitory disulphide with cJun C269. Conversion of this cysteine to an electrophile generates an irreversible and selective cJun inhibitor, validating this approach for covalent antagonist discovery.

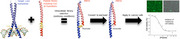

## 1 Introduction

Peptides are increasingly asserting their utility as therapeutic lead molecules to target challenging intracellular protein targets.^[^ ^1^ ^]^ The effectiveness of peptides in targeting protein interaction surfaces is now well established due to their ability to bind to broader and shallower interfaces than small molecules, with enhanced binding affinity and selectivity. Although this is also an advantage for larger biologics such as antibodies, peptides are preferable due to their lower synthetic cost and immunogenicity, as well as their potential to penetrate cells and access intracellular targets. Prominent work in the field has developed cyclization strategies to react combinations of peptide head/sidechain/tail together toward ordering and stabilizing the structure, aiding with metabolic stability and target affinity.^[^ ^2^, ^3^ ^]^ Like small molecules, it is also possible to covalently attach peptides to target proteins using a range of biocompatible chemistry.^[^ ^4^, ^5^, ^6^ ^]^ In doing so, target proteins can be rendered inactive by a selective reaction with a nucleophilic residue that blocks a site essential for binding or required for catalysis, with the protein remaining inactive until its replacement by the production of a new copy.

**Figure 1:**
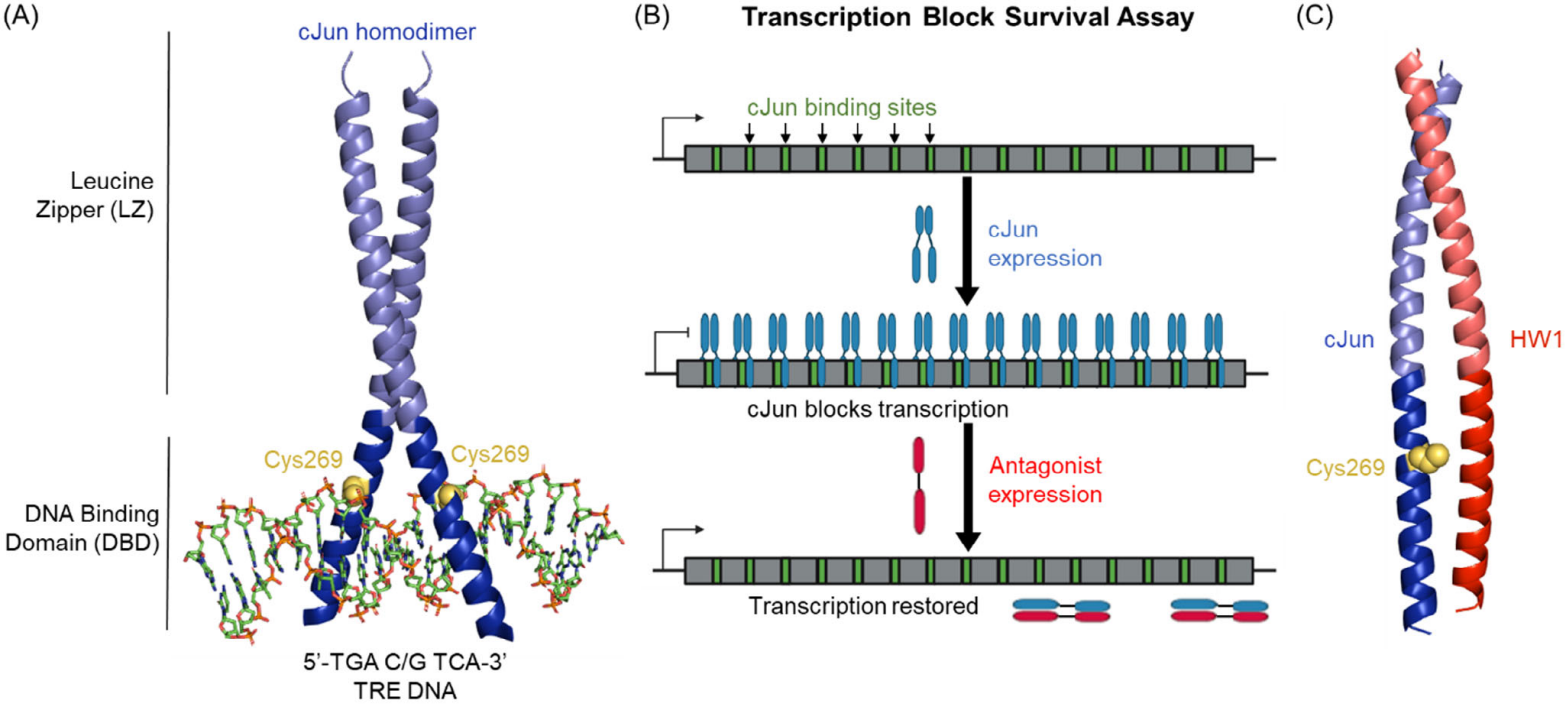
Structural detail and schematics of cJun, HW1, and the screening approach. A) Crystal structure of the bZIP domain of cJun bound as a homodimer to TRE DNA, with C269 shown as yellow spheres (PDB: 2H7H). B) Cartoon schematic describing the TBS assay whereby cJun binding sites are incorporated into the coding region of an essential gene, which prevents its transcription when cJun is bound. The TBS assay readout is cell growth, which is abrogated when cJun is bound, but antagonism by a peptide library member restores cell growth. C) Alphafold‐multimer (using the Google Colab platform, v.2.1.) prediction of the interaction between cJun and its TBS‐derived antagonist HW1.^[^ ^15^ ^]^ pTM score: 0.68; ipTM score: 0.72; pLDDT illustrated in Figure S1 (Supporting Information).

Peptide antagonists can be used to inhibit oncogenic transcription factors (TFs) that possess targetable protein–protein interactions (PPI) as well as protein‐DNA interaction surfaces.^[^ ^7^ ^]^ The broad PPI interfaces of intracellular TFs are ideal candidates for peptide targeting, with oncogenic signals able to be ablated directly at the transcriptional level, counteracting dysregulation at upstream points in the signalling cascade. One such target is cJun; a member of the AP‐1 family of dimeric transcription factors that is overexpressed and upregulated in patient tumor samples in specific cancer types.^[^ ^8^ ^]^ Further, validation in cancer model systems indicates that cJun inhibition can effectively inhibit tumor progression and proliferation.^[^ ^9^, ^10^ ^]^ By binding to 12‐O‐tetradecanoylphorbol‐13‐acetate (TPA) response element (TRE) DNA sites (5′‐TGA C/G TCA‐3′), cJun regulates the expression of genes such as CyclinD1, MMP9, and FasL that are involved in differentiation, proliferation, and apoptosis. Therefore, increased cJun activity can produce hallmark cancer cell behaviors.^[^ ^11^ ^]^ cJun binds DNA as a dimer via its bZIP domain. This is comprised of a leucine zipper (LZ) coiled coil dimerization domain with a cationic N‐terminal DNA binding domain (DBD) which scissor grips the DNA major groove to form specific contacts with the TRE DNA bases (**Figure** **1**A).^[^ ^12^ ^]^

**Figure 2:**
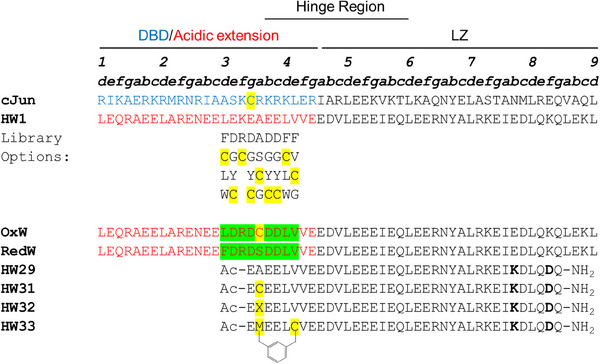
Peptide sequences and the library design used in the TBS screen and subsequent antagonist optimization. The target cJun and previously derived antagonist HW1 sequences are shown.^[^ ^13^ ^]^ HW1 is shown without terminal modifications as this represents the in vivo peptide library, but this peptide is N‐terminally acetylated and C‐terminally amidated when tested in vitro. The library options are shown below their positions in the HW1 backbone, e.g. position **d3** is randomized between Phe, Cys, Leu, Trp, etc. OxW and RedW are the winner peptides selected in SHuffle Express and BL21 Gold cells, respectively. HW29 is an optimized peptide antagonist from our previous work, with a lactam bridge between the K and D residues in bold.^[^ ^3^ ^]^ HW31‐HW33 are the covalent antagonists investigated in this study, with X representing a dehydroalanine residue and the electrophilic warhead crosslink shown on HW33, formed by a bisalkylation reaction between Met2 and Cys6.

We previously developed cJun peptide antagonists using a Transcription Block Survival (TBS) assay (Figure 1B).^[^ ^13^ ^]^ This intracellular peptide library screening platform moves beyond conventional peptide screening systems by selecting peptides based on their ability to not only bind to the target but to meet the more demanding task of functionally antagonizing it by i) binding and, more importantly, ii) blocking its ability to form protein‐DNA interactions. First, TRE sites required for cJun binding were incorporated into the coding region of an essential gene, dihydrofolate reductase (DHFR). By selective inhibition of bacterial DHFR using trimethoprim (TMP), *E. coli* are rendered dependent on the expression of exogenous TRE‐mouse DHFR (mDHFR) from a transformed plasmid. mDHFR is uninhibited by TMP at the concentrations used. The introduction of a cJun expression plasmid causes a transcriptional block within the essential TRE‐mDHFR gene, functioning as a roadblock to sterically hinder RNA polymerase. This prevents the transcription of the essential protein, abrogating cell growth. Only concurrent expression of functional cJun antagonists sequesters cJun, prevents DNA binding, and facilitates TRE‐mDHFR transcription and translation. Therefore, cell growth correlates with cJun/TRE peptide antagonist efficacy to provide a genotype‐to‐phenotype readout that enables competition library screening. TBS was then used to screen a peptide library, identifying HW1 as a 210 pM affinity cJun antagonist (Figures 1C and 2), which was subsequently optimized by downsizing and *i*→*i+4* sidechain‐to‐sidechain cyclization via a K→D lactam bridge to produce HW29.^[^ ^3^, ^13^ ^]^

Importantly, cJun contains an exposed Cys residue (C269) within its DNA‐binding domain. This forms part of the protein‐DNA interaction surface and is flanked by residues that form direct hydrogen bonds with TRE DNA bases (Figure 1A). Previous reports have indicated that C269 is a redox switch capable of turning off cJun DNA binding upon disulphide formation.^[^ ^14^ ^]^ Here, we describe the generation of a peptide library to identify a position to incorporate a Cys in the antagonist peptide to facilitate effective disulphide bond formation with cJun C269. The selected Cys was next replaced with an electrophilic covalent warhead to provide potent, irreversible, and selective inhibition of cJun function.

## 2 Results

### 2.1 Covalent‐TBS Screening Identifies Appropriate Cys Positioning to form an Inhibitory Disulphide Bond with the Target

To design the genetically encoded peptide library, we considered amino acid positions that were most likely to be in proximity to cJun C269 upon binding. Without experimental structural information on the interaction between HW1 and cJun, the DNA‐bound cJun homodimer structure and an Alphafold‐multimer generated prediction of the complex were used to provide insight (Figure 1A,C). The Alphafold‐multimer prediction indicated that the peptides form a coiled coil interaction across their full length. There are some reservations about the validity of the structure owing to the unusual packing of charged residues in the typically hydrophobic core of this interaction in the DBD/acidic extension region, as well as the lower confidence in the prediction of this region (Figure S1, Supporting Information). Nonetheless, we used the prediction to determine a broad section within the HW1 sequence that is likely to be held in close proximity to cJun C269. We wanted to generate a peptide library that would allow for the selection of either a cysteine residue or the original amino acid at each of these positions. Therefore, the genetically encoded peptide library contained nine consecutive semi‐randomized positions allowing for the selection of i) the original amino acid, ii) Cys, or iii) up to two additional options, which were not required but were necessary owing to mixed‐codon degeneracy during cloning (**Figure** **2**). In the case of parental Glu or Lys, this would produce an unnecessarily large library, but replacing with Asp or Arg options instead limited this. This afforded a covalent‐library of 131072 members, which was produced using the cloning methodology described previously^[^ ^13^ ^]^ and in which the vast overrepresentation of colony numbers upon transformation ensured library coverage (>99.9% probability of full library coverage).

The TBS library screening platform was introduced into two *E. coli* strains: BL21‐Gold and SHuffle Express, to probe the same library in reducing versus non‐reducing cytoplasmic environments, respectively. We previously deployed an analogous approach to introduce a conformational constraint into a target protein during library screening.^[^ ^16^ ^]^ SHuffle Express cells are a BL21‐derived strain engineered to promote disulphide bond formation in the cytoplasm as they lack thioredoxin reductase and glutathione reductase and constitutively express the disulphide isomerase DsbC. The library was then transformed into these two systems, and after ten passages in SHuffle Express cells and six passages in BL21‐Gold cells, two overall library winner peptides were selected: OxW and RedW (Figure 2; Figure S2, Supporting Information). The approach was validated by a selection of Cys at ***a3*** (C19) within the OxW sequence, with no Cys selected within the reducing cell line, implying that Cys incorporation at the ***a3*** position had increased cJun antagonism due to the formation of a cJun‐OxW disulphide bond. In OxW, all other positions selected the parental amino acid, i.e., within the template sequence or the structurally similar option. Reassuringly, the RedW sequence was almost identical to OxW, suggesting that an effective non‐covalent binder had been identified in both cell lines while confirming that under reducing conditions, Cys options provide no advantage to binding. We note that one larger hydrophobic residue, Phe, was selected at position **d3** in RedW rather than the parental Leu, but this has not been further studied.

### 2.2 Convergence of Studies Produced an Optimized Peptide Antagonist Design

The work presented here was undertaken in parallel with our previously published study in which HW1 was downsized and lactamized,^[^ ^3^ ^]^ as two divergent routes toward peptide optimization. In particular, we determined the maximum viable truncation of the parent peptide and combined it with systematic lactamization to identify a position near the C‐terminus of the truncated peptide as the optimal position in which to incorporate an i→i+4, K→D intramolecular lactam bridge. This was found to induce helicity, target binding, and serum stability in the peptide. Combining insight gained from that study with the results of the covalent‐TBS screen described here, a 36‐mer peptide was synthesized (HW31, Figure S3, Supporting Information) with the optimized lactam side chain cyclization and with a Cys at the position selected in the screen.

### 2.3 HW31 Forms a Disulphide Bond with cJun to Provide Improved Target Antagonism

**Figure 3:**
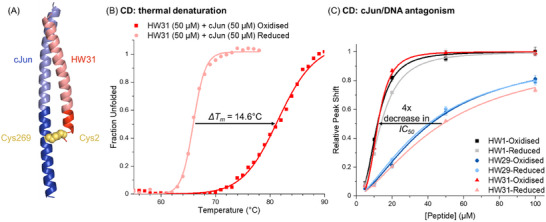
HW31 covalently attaches to target cJun C269 to improve antagonism. A) Alphafold 3 prediction of the structure of the cJun/HW31 complex, which indicates the formation of a disulphide between cJun C269 and HW31 C2.^[^ ^17^ ^]^ pTM score: 0.75; ipTM score: 0.77; pLDDT illustrated in Figure S6 (Supporting Information). B) CD thermal denaturation curves (following the signal at 222 nm) of the cJun/HW31 heterodimer under reducing and oxidizing conditions show a higher T_m_ in oxidizing conditions, indicative of increased binding affinity. C) CD cJun/TRE DNA antagonism data whereby the signal corresponding to the TRE DNA structure is monitored and its relative shift from bound to unbound at varying peptide concentration is determined. Triplicate data were fitted to a Hill equation (OriginPro). Comparing oxidizing/reducing conditions illustrates the improved antagonism efficacy of HW31 in oxidizing conditions due to the formation of an intermolecular disulphide with the target cJun C269, improving its activity to that of HW1. HW1 and HW29 show no significant change in antagonism between oxidizing and reducing conditions. Data for all determined T_m_ and IC_50_ values are found in Table S1 (Supporting Information).

A mixture of HW31 (50 µm) and cJun (50 µm, Figure S4, Supporting Information) was first shown to form a disulphide‐bridged complex by ESI‐MS, which could be reduced back to the separate species by the addition of TCEP (Figure S5, Supporting Information). Further, an Alphafold 3 prediction of HW31 (prediction without lactam bridge) bound to cJun indicates the complex forms the predicted coiled coil interaction with the formation of a disulphide between cJun C269 and HW31 C2 (**Figure** **3**A; Figure S6, Supporting Information).^[^ ^17^ ^]^ The interaction was next characterized using circular dichroism (CD) experiments in both oxidizing and reducing conditions. The mixing of HW31 and cJun in both conditions provided a clear increase in helicity, indicative of a binding interaction (Figure S7, Supporting Information). The measured HW31‐cJun heterodimer complex was ≈10% more helical in the oxidized buffer relative to the reduced. Further, thermal denaturation experiments on the heterodimeric mixture showed a shift in *T_m_* from 66.6 °C in reducing buffer to 81.2 °C in oxidizing buffer (*ΔT_m_* = 14.6 °C), most likely due to the formation of the disulphide bond (Figure 3B). Analytical size exclusion chromatography further validated HW31/cJun binding and confirmed the formation of a 1:1 stoichiometric complex (Figure S8, Supporting Information).

CD antagonism experiments were next carried out, as previously described,^[^ ^13^, ^18^ ^]^ whereby the structure of a TRE DNA duplex was monitored by following a peak at 281 nm, which shifts upon binding to cJun. The introduction of antagonist to the sample causes the peak to shift back to its unbound signal in a dose‐dependent manner as the cJun is sequestered, leaving unbound DNA in its original structure (Figure S9, Supporting Information). As there is no protein absorbance in this region of the CD spectrum, this measures DNA structure only and produces a simple readout of the proportion of free/cJun‐bound DNA at different antagonist concentrations. By fitting these plots to a Hill equation (OriginPro), *IC_50_* values for HW1, HW29, and HW31 were determined in both reducing and oxidizing conditions (Table S1, Supporting Information; Figure 3C). These experiments indicate a 4‐fold improvement in antagonism for HW31 for oxidizing relative to reducing conditions, which, together with MS data, demonstrates that disulphide bond formation with cJun C269 enhances antagonism. By contrast, HW1 and HW29, which both lack Cys, exhibit the same *IC_50_* in reducing or oxidizing conditions. The *IC_50_* of HW29 was the same as HW31 under reducing conditions, indicating that the single Ala to Cys change has no significant effect unless covalently bound. The reduction in activity caused by truncating 57‐mer HW1 to 36‐mer peptide HW31 was fully restored upon lactam bridge and disulphide bond formation.

### 2.4 Converting HW31 Into an Irreversible Covalent Antagonist

**Figure 4:**
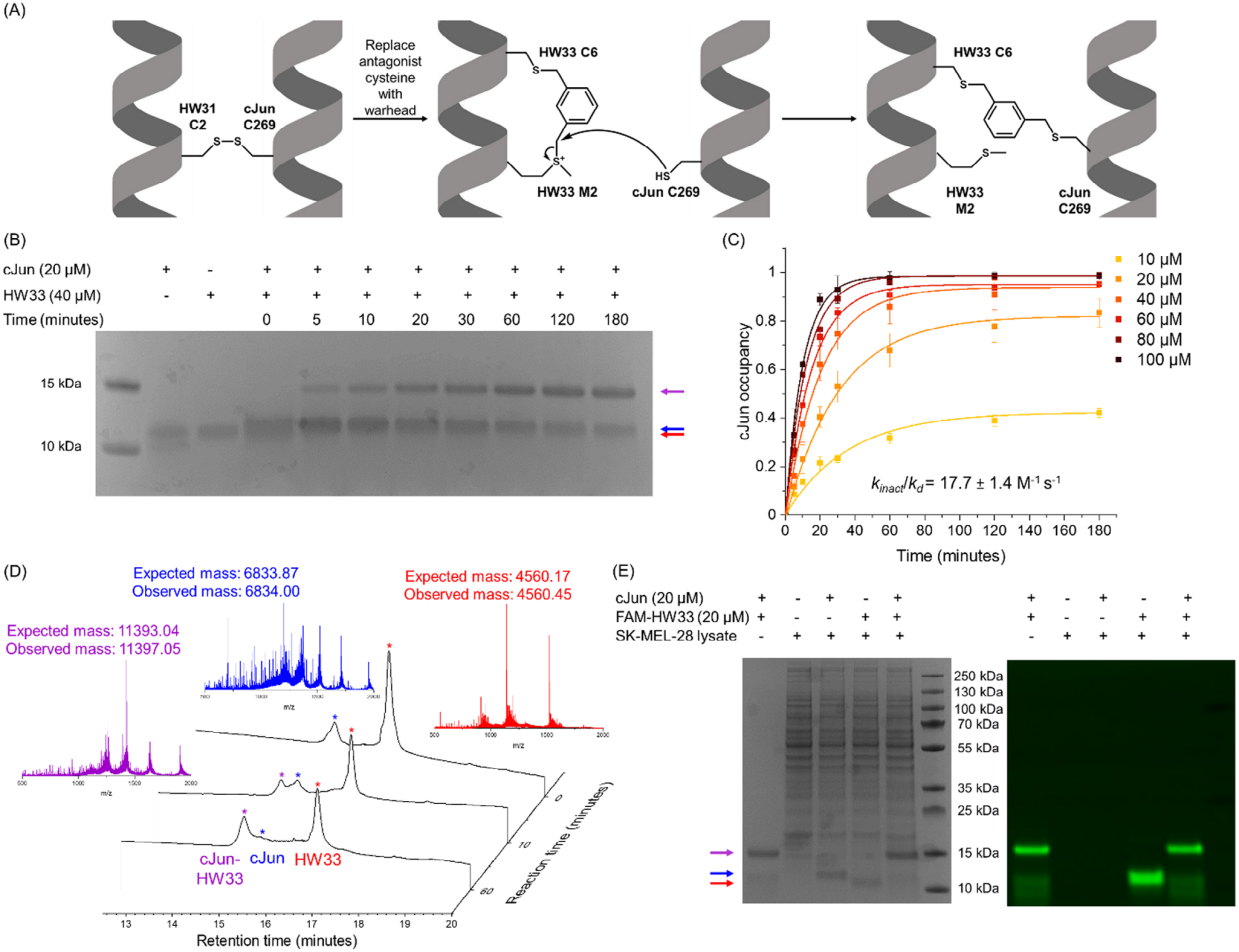
HW33 reacts rapidly and selectively with cJun. A) Schematic showing the conversion of the disulphide forming peptide HW31 to the bisalkylated, irreversible covalent inhibitor HW33 and its reacted linkage with cJun. B) SDS‐PAGE analysis of the reaction between cJun (20 µm, MW = 6.8 kDa) and HW33 (40 µm, MW = 4.6 kDa) over 3 h, which shows the formation of the covalently linked product (MW = 11.4 KDa), with some unreacted cJun and HW33 still present. The higher apparent molecular weights of these peptides (compared to their MS‐confirmed masses) are likely due to their high charge density, which alters SDS binding and charge‐to‐mass ratio.^[^ ^20^ ^]^ Incubation with CREB1 produced no covalent product after 24 h (Figure S16, Supporting Information). Uncropped image from 4B is shown in Figure S13 (Supporting Information). C) cJun (20 µm) occupancy upon reaction with HW33 (10–100 µm) monitored by LC‐MS. Data plotted over time for each concentration and fitted to a single exponential equation to give a kobs for each peptide concentration tested. D) Selected LC‐MS traces during the reaction of cJun (20 µm) with HW33 (40 µm). E) SDS‐PAGE analysis of the reaction between cJun (20 µm) and FAM‐HW33 (20 µm) after 24 h incubation in SK‐MEL‐28 cell lysate, which shows the selective reaction with the cJun bZIP and no significant observed promiscuous reaction.

Having determined the antagonist Cys position most suitable for forming a covalent interaction with cJun C269 and demonstrating that disulphide formation improves cJun antagonism, further work was needed to develop this into an irreversible inhibitor. To do so, two methodologies were adopted. Firstly, the cysteine was converted to dehydroalanine using a reduction‐elimination reaction.^[^ ^19^ ^]^ HW31 was reacted with Ellman's reagent (DTNB) to form the disulphide, which was purified and subsequently reacted with tris(dimethylamino)phosphine (HMPT) to give the dehydroalanine‐containing peptide HW32 (Figure 2; Figure S10, Supporting Information). The second covalent methodology utilised a bis‐alkylation reaction between an *i* to *i+4* methionine and cysteine pair using meta‐dibromomethylbenzene (mDBMB). This sidechain‐to‐sidechain crosslinking reaction produces a sulfonium on the methionine residue, which has been shown to undergo proximity‐induced nucleophilic substitution with a target cysteine.^[^ ^5^ ^]^ The met and cys pair was introduced into the HW31 sequence with the methionine placed at the cysteine position of HW31 and the cysteine placed at an *i+4* position to give HW33 (Figures 2 and **4**A; Figure S11, Supporting Information).

HW32 and HW33 (40 µm) were next incubated with cJun (20 µm) at 37 °C, and the conjugation reactions were monitored by SDS‐PAGE (Figure 4B; Figures S12 and S13, Supporting Information). A fold excess of antagonist peptide was investigated to increase the reaction rate such that both peptides were observed to react, allowing comparison. HW33 was found to be significantly more reactive than HW32, with the conjugated product observed at the first timepoint of 5 min and the reaction approaching completion after 3 h. The dehydroalanine‐containing HW32 was significantly less effective with the product first observed after 2 h, and the unreacted cJun band was still visible after 48 h. The reaction kinetics of HW33 were therefore more thoroughly studied across a range of peptide concentrations (10–100 µm) by LC‐MS to determine the *k_inact_*/*k_d_* as 18 ± 1 M^−1^ s^−1^ (Figure 4C,D; Figure S14, Supporting Information). Lower peptide concentrations may have produced a higher value for this parameter, but they could not be assessed using LC‐MS for detection.

**Figure 5:**
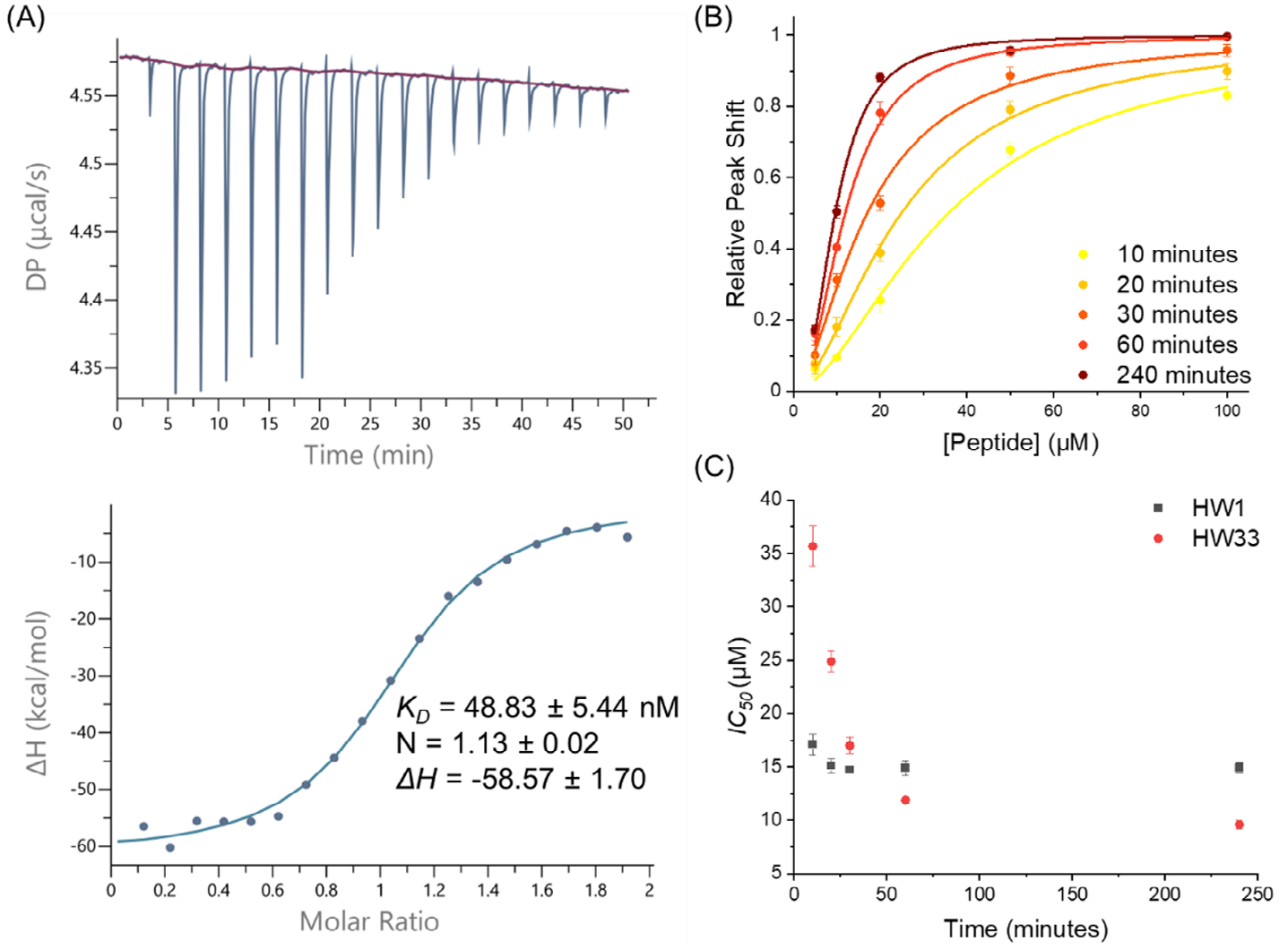
Biophysical characterization of HW33 binding and antagonizing cJun. A) ITC data for HW33 (20 µm) titrated into cJun (2 µm) at pH 6.5 to remove the covalent component and study non‐covalent binding only. The raw power compensation plot is shown in the upper graph, and the integrated data points and single site model fitted line are shown in the lower graph. The thermodynamic parameters shown are averages from three experiments. B) CD antagonism plots for HW33 over time showing an increase in antagonist efficacy. Triplicate data were fitted to a Hill equation to determine IC_50_ values using OriginPro. C) IC_50_ from CD antagonism experiments plotted over time for both HW1 and HW33 illustrate a time dependence on HW33 efficacy but not HW1, with HW33 showing improved antagonism over HW1 after 60 min.

The binding affinity for the interaction between HW33 and cJun was also determined by isothermal titration calorimetry to be 49 ± 5 nM, compared to the previously determined affinity for HW29 of 87 ± 17 nM (**Figure** **5**A).^[^ ^3^ ^]^ This experiment was performed at pH 6.5 as a measure of the non‐covalent binding component. The *IC_50_* of HW33 was also determined and compared to HW1 in a CD antagonism experiment over a 4 h time course, which showed a 5 fold increase in antagonism for HW33 over that time (Figure 5B,C). This demonstrates time‐dependent inhibition of cJun by HW33, which is further indicative of covalent inhibition. After 4 h, HW33 exhibited the lowest *IC_50_* observed from this series of peptides at 10 ± 0.4 µm. HW1 exhibits a consistent *IC_50_* averaging at 13 µm, with the 10 min time point showing a slight increase in value, which indicates that at this time‐point, the system had not equilibrated. This result was further validated by fluorescence polarization, which also indicated a time‐dependent *IC_50_* value for HW33, producing improved cJun/TRE DNA antagonism compared to HW1 after 240 min (Figure S15, Supporting Information). The cJun/TRE DNA antagonism of HW33 occurs at a slower rate in this experiment due to the lower component concentrations used.

### 2.5 Covalent Inhibition by HW33 is Highly Selective

The selectivity of this reaction, which is highly proximity‐driven, was first investigated by incubating HW33 (40 µm) with CREB1 (20 µm), a closely related bZIP‐containing TF that contains a Cys residue at the same position within the bZIP domain as cJun. Moreover, CREB1 contains two additional Cys residues located within the LZ domain that are not found within cJun. The reaction with CREB1 was monitored by SDS‐PAGE, with no conjugation observed after 24 h (Figure S16, Supporting Information), providing further evidence that this covalent conjugation is selectively driven by proximity due to the PPI. To further investigate HW33 selectivity for cJun, a reaction was next undertaken in SK‐MEL‐28 human melanoma cell lysate using FAM‐HW33, in which the N‐terminal acetyl group of HW33 was replaced with carboxyfluorescein to allow for peptide detection (Figure S17, Supporting Information). Samples containing cJun bZIP (20 µm) and FAM‐HW33 (20 µm) were incubated with or without lysate (10 µg) at 37 °C for 4 h before analysis by SDS‐PAGE (Figure 4E). The reaction without the cell lysate confirmed that fluorescent labeling did not reduce peptide reactivity. Incubation of the peptide alone with the lysate showed a single band of unreacted FAM‐HW33 (red arrow), indicating that the peptide did not significantly react with free thiols in the proteome. The reaction between the peptide and supplemented cJun (blue arrow) in the cell lysate proceeded to produce the FAM‐HW33‐cJun covalently attached product (purple arrow). This confirms the proximity‐induced nature of the covalent target attachment as the tight interaction with cJun is required for the reaction to proceed.

### 2.6 FAM‐HW33‐NLSTAT Enters Melanoma Cells in Culture, Depletes cJun, and Reduces Cell Viability

**Figure 6:**
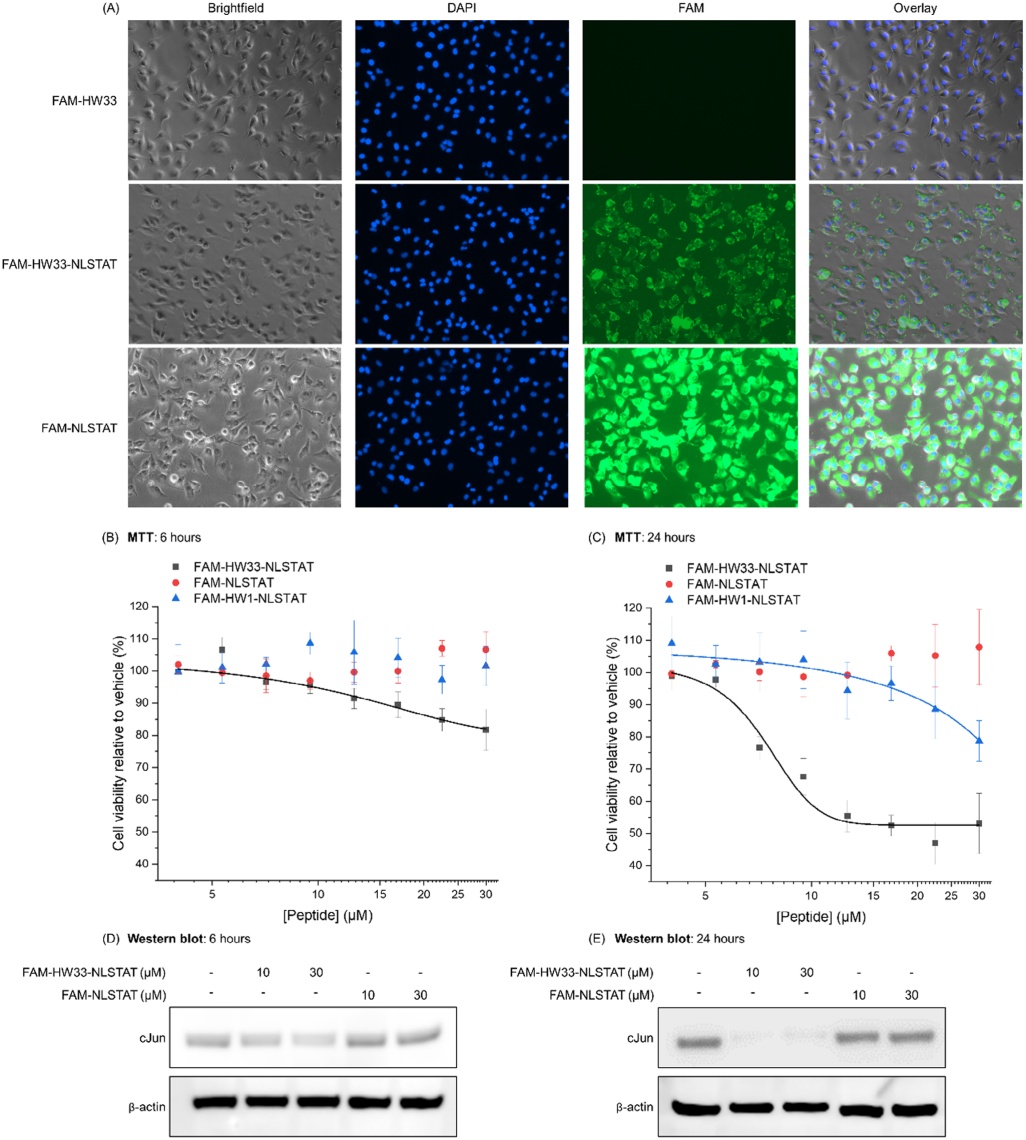
FAM‐HW33‐NLSTAT enters melanoma cells, resulting in both cJun depletion and reduced cell viability. A) Imaging of SK‐MEL‐28 cells treated with 30 µm peptide for 6 h at 37 °C shows that NLSTAT is required to transport FAM‐HW33‐NLSTAT into cells, with FAM‐HW33 showing no evidence of uptake. FAM‐NLSTAT enters cells effectively and is evenly distributed in the cytosol, with neither this nor FAM‐HW33‐NLSTAT showing significant colocalization with the nucleus. SK‐MEL‐28 cell viability was measured via MTT assay upon treatment with a range of FAM‐HW33‐NLSTAT, FAM‐NLSTAT, or FAM‐HW1‐NLSTAT concentrations at B) 6 h and C) 24 h, showing that the HW33 cargo is required for loss of cell viability, which it imposes in a time‐dependent manner. The covalent HW33 cargo is significantly more effective at reducing cell viability than HW1. Data are normalized to vehicle control and are the average of three independent experiments, with error bars shown as one standard deviation and fitted to the dose response equation using OriginPro. Western blot analysis of cJun (and β‐actin control) levels in SK‐MEL‐28 cells treated by indicated peptide concentrations for D) 6 h and E) 24 h. Images are representative of three independent experiments. Uncropped western blot images are shown in Figures S22 and S23 (Supporting Information).

Inhibition of cJun has been shown to suppress the growth of SK‐MEL‐28 malignant melanoma cells and was, therefore, used here to test peptide efficacy in cellular assays.^[^ ^10^, ^21^ ^]^ Due to the size and charge of FAM‐HW33, a cell penetrating peptide (CPP) comprised of residues 48–57 of the HIV‐1 TAT protein^[^ ^22^ ^]^ and a nuclear localization signal (NLS) from the SV40 large T‐cell antigen^[^ ^23^ ^]^ were appended to the C‐terminus of the molecule (NLSTAT: PKKKRKVYGRKKRRQRRR) to produce FAM‐HW33‐NLSTAT for use in cell‐based assays (Figure S18, Supporting Information). FAM‐HW33‐NLSTAT, FAM‐HW33, and FAM‐NLSTAT peptides (Figure S19, Supporting Information) were applied to SK‐MEL‐28 cells at 10 and 30 µm, and the cells were fixed and counterstained with DAPI before imaging (**Figure** **6**A; Figure S20, Supporting Information). FAM fluorescence was observed within the cells at both concentrations for the NLSTAT‐containing peptides, but no fluorescence was observed for FAM‐HW33, indicating that the addition of the CPP tag is an absolute requirement for FAM‐HW33‐NLSTAT uptake. Surprisingly, neither FAM‐HW33‐NLSTAT or FAM‐NLSTAT appeared to significantly localise within the nucleus, although some fluorescence can be observed there. Additionally, enhanced fluorescence was observed for FAM‐NLSTAT relative to FAM‐HW33‐NLSTAT, with the specific localization appearing to differ, indicating that the HW33 cargo is affecting the CPP pathways into and within the cell. No significant entry was observed for FAM‐HW1‐NLSTAT at 10 µm, with a similar degree of entry and localization observed for 30 µm FAM‐HW1‐NLSTAT as for FAM‐HW33‐NLSTAT.

Having confirmed FAM‐HW33‐NLSTAT entry into cells, MTT assays were performed to assess cell viability upon treatment with FAM‐HW33‐NLSTAT, FAM‐NLSTAT, and FAM‐HW1‐NLSTAT (4–30 µm) at 6 and 24 h (Figure 6B,C). At 24 h, SK‐MEL‐28 cells treated with 30 µm FAM‐HW33‐NLSTAT displayed 53% cell viability compared to vehicle‐treated cells with an *IC_50_* of 7 ± 0.5 µm, and with no significant effect observed for the FAM‐NLSTAT alone. Treatment with 30 µm FAM‐HW1‐NLSTAT reduced cell viability to 79% at this time point, and this significantly lower activity prevented the determination of an *IC_50_* value within the assay window. The loss of cell viability was also shown to be time‐dependent, as the earlier time point of 6 h showed that cells treated with 30 µm FAM‐HW33‐NLSTAT displayed 82% viability compared to vehicle treatment. Both FAM‐NLSTAT and FAM‐HW1‐NLSTAT produced no significant effect at this time point. No effect on cell viability was observed upon treatment with FAM‐HW33, which is unable to enter cells (Figure S21, Supporting Information). A longer assay window would likely have produced further reductions in cell viability, but as there were clear and consistent decreases in viability at 24 h this was not tested.

To further study the effect of the peptide upon cJun, cells were next treated with 10 or 30 µm FAM‐HW33‐NLSTAT or FAM‐NLSTAT for either 6 or 24 h and then collected, with cJun protein levels determined by western blot (Figure 6D,E; Figures S22–S24, Supporting Information). FAM‐HW33‐NLSTAT treatment resulted in a dose‐dependent decrease in cJun levels. At 6 h, 10 µm peptide reduced cJun levels to 51%, while at 30 µm, only 22% of cJun was observed relative to vehicle controls. After 24 h, almost complete depletion of cJun was observed, with only ≈11% of the amount observed upon vehicle treatment at both treatment concentrations. This cJun depletion effect was also observed in cell lysate upon the addition of FAM‐HW33‐NLSTAT, with the addition of a protease inhibitor cocktail (Figure S25, Supporting Information). These experiments indicate that although FAM‐HW33‐NLSTAT does not appear to predominantly localise at the nucleus after 6 h, it is nonetheless highly effective at targeting cJun while imparting a significant impact on cell viability.

## 3 Discussion

We describe the deployment of an intracellular TBS library screen performed under oxidizing cytoplasmic conditions to develop covalent inhibitors of protein‐DNA interactions. We demonstrate that Cys residues for intermolecular disulphide formation with a target Cys can be selected within a TBS screen using Shuffle Express cells. The approach probes for Cys residues within a target protein that are amenable to covalent inhibition, where this covalent linkage antagonizes function. Our work demonstrates that when the same library is screened under reducing conditions, where there is no selective pressure for their enrichment, Cys residues are not selected. However, high sequence similarity throughout the remainder of the scrambled region indicates that high affinity non‐covalent antagonists are enriched under both oxidizing and reducing conditions. In this work, the potential for increased antagonist efficacy by covalently targeting Cys269 within the DBD of cJun has been confirmed. This result was anticipated, hence its use as a model system here, but not all TF/DNA interactions are so thoroughly structurally studied as cJun/TRE, and not all cysteines can be covalently modified for increased antagonism. Moreover, the location of the antagonist cysteine is not readily rationally predicted. Consideration of typical coiled coil interactions, via helical wheel projection, suggests that multiple positions in the antagonist are in proximity to cJun C269, and a peptide with a covalent warhead at each would typically be synthesized and tested individually.

This proof‐of‐concept work shows that sites for covalent inhibition can be identified within the screening protocol. The approach opens the possibility to screen naïve libraries containing many Cys options against less‐studied targets. This will select for a peptide sequence capable of functionally antagonizing the target TFs interaction with its consensus DNA sequence, and Cys residues identified within the antagonist will be amenable to further optimization to incorporate electrophiles. In this work, two electrophiles were tested and shown to react, with varying rates, but a range of moieties are known with Cys selectivity, which could be used.^[^ ^6^ ^]^ Covalent warheads should be weak electrophiles that require a proximity induction of the reaction in order to be applied as therapeutics to eliminate off‐target effects. As such, the increased binding selectivity produced by screening within the *E. coli*. cytoplasm is clearly beneficial. The high‐affinity and selective binding of HW33 to cJun results in selective covalent inhibition, with incubation of the peptide alone at 20 µm in melanoma cell lysate producing no significant reaction across the proteome and the highly related CREB1 protein, with three Cys residues, being unreactive with the antagonist. This high‐affinity target binding also leads to favorable kinetics, with the product observed within 5 min of incubation in vitro. We also observed the improved antagonism that this reaction produces as the ability of HW33 to remove cJun from TRE DNA was shown to increase as the reaction proceeded.

Crucially, the result of this work, FAM‐HW33‐NLSTAT, was shown to reduce SK‐MEL‐28 melanoma cell viability through cJun depletion, validating the utility of this TBS workflow toward therapeutic antagonists of protein‐DNA interactions. Work by Guo et al., in SK‐MEL‐28 cells, has shown that a decrease in cJun levels was produced using a TRAF6 inhibitor, which led to an induction of autophagy and apoptosis through reduced ATG16L2 expression.^[^ ^21^ ^]^ cJun depletion was also observed upon treatment with cJun inhibitor Ailanthone in SK‐MEL‐28 cells by Yu et al., and subsequently shown to be restored by a proteasome inhibitor, MG132, indicating that target binding was inducing proteasomal degradation.^[^ ^10^ ^]^ However, in their experiments and an equivalent performed by us using FAM‐HW33‐NLSTAT, proteasome inhibitor treatment alone leads to an increase in detected cJun, so it is difficult to draw a conclusion from this. The reaction of FAM‐HW33‐NLSTAT with cJun may be leading to an increase in cJun degradation, but it remains possible that some other, perhaps regulatory, mechanism is reducing the amount of cJun detected. Western blotting of cJun was expected to show a band shift upon treatment with FAM‐HW33‐NLSTAT corresponding to the covalent complex, but as this was not observed, it may indicate that cJun degradation occurs rapidly upon reaction, and as such, no observable amount of covalent complex is maintained. This will form part of the further study and optimization of this peptide. Although the route to cJun depletion has not yet been fully explored, it is clearly occurring in a dose‐ and time‐dependent manner and is unequivocally observed after only 6 h.

It was anticipated that the NLSTAT peptide would lead to significant nuclear localization,^[^ ^22^, ^23^ ^]^ as has been widely reported in the literature however, this was not the case even in the absence of the HW33 cargo. This may be a cell type‐specific effect, as we have not been able to identify other work using this CPP with SK‐MEL‐28 cells. The observation of FAM punctate in FAM‐HW33‐NLSTAT treated cells, which was not observed in FAM‐NLSTAT treated cells, could also be explored in future work as this may indicate the peptide is trapped within endosomes.^[^ ^24^ ^]^ However, importantly, neither nuclear entry nor observation of punctae precluded HW33 impact on cell viability. This intracellular delivery method could be optimized in future work by exploration of alternative CPPs^[^ ^25^ ^]^ or other methods such as liposome encapsulation,^[^ ^26^ ^]^ although since the peptide is highly effective without nuclear localization it is possible that this is not needed, and that covalent cJun inhibition occurs outside of the nucleus immediately following protein translation.

This work represents a significant step in expanding the possibilities of screening for covalent inhibition of protein/protein and protein/DNA interactions. In a single screening process, with appropriate library design, we are now capable of identifying potent TF inhibitors and determining appropriate Cys sites on the target protein that are amenable to covalent inhibition. Crucially, as this occurs within the Covalent‐TBS assay, only bridging of Cys residues where covalent inhibition produces enhanced antagonism will be identified, producing a streamlined process of covalent antagonist development. Since TBS screening occurs within *E. coli* cells, hits will tend to exhibit higher selectivity and biostability and lower toxicity. Further, TBS is significantly more sustainable and cost‐effective than equivalent synthetic screening approaches. The lead molecule produced in this work, FAM‐HW33‐NLSTAT, combines two avenues of research on *i)* downsizing and lactamization of a TBS‐selected peptide and *ii)* the introduction of a covalent warhead to produce an optimized bicyclic peptide. FAM‐HW33‐NLSTAT has high‐affinity yet selective binding to cJun, allowing for efficient reaction of the warhead with cJun C269. The peptide enters cancer cells and is effective at antagonizing the oncogenic interaction of cJun with TRE DNA to reduce melanoma cell viability. The utility of this covalent screening approach has been clearly demonstrated by the significant improvement in the efficacy of HW33 relative to HW1 in cell‐based assays, with 28% of the peptide truncated. FAM‐HW33‐NLSTAT is able to enter melanoma cells more effectively, at lower concentrations, and produced a 2.2x larger reduction in cell viability at 30 µm.

## 4 Conclusion

In summary, we utilized an intracellular TBS library screen performed under oxidizing cytoplasmic conditions for the development of covalent inhibitors. Our covalent library contained 9 Cys options, with only one selected in oxidizing conditions, and remarkable hit sequence similarity when screened under reducing conditions where no Cys residues were selected, providing robust evidence for non‐covalent interaction prior to disulphide formation and an increase in antagonist efficacy upon disulphide formation. An optimized disulphide‐forming peptide (HW31) was 37% smaller than the HW1 design template with the same efficacy in oxidizing conditions. This further confirms the effectiveness of TBS in deriving peptides able to release TRE DNA from cJun. Next, we replaced Cys with a mDBMB‐based warhead (HW33) to react irreversibly with cJun C269 in a time and concentration‐dependent manner. Selectivity was demonstrated via incubation with bZIP homologue CREB1, with no evidence of covalent product despite exposure to three alternative target Cys residues, including one at the analogous position within cJun. This provided increased evidence that the interaction is first driven by selective PPI formation for appropriate positioning of the warhead, followed by the desired reaction with C269. Finally, we demonstrated that the downsized warhead‐containing peptide HW33 can be modified to penetrate SK‐MEL‐28 melanoma cells, where it depletes cJun and reduces cell viability. Overall, this covalent selection approach significantly expands upon the utility of the TBS system to determine the feasibility of covalent inhibition of target proteins.

## Conflict of Interest

The authors declare no conflict of interest.

## Author Contributions

A.B. conducted the experiments, whilst all authors contributed to the experimental design. J.M.M. directed the research. All authors participated in the data analysis and writing of the paper.

## Supporting information



Supporting Information

## Data Availability

The data that support the findings of this study are available from the corresponding author upon reasonable request.
