# Supplementary material for: An Intracellular Peptide Library Screening Platform Identifies Irreversible Covalent Transcription Factor Inhibitors
Source: Adv Sci (Weinh). 2025 Mar 17;12(18):2416963. doi: 10.1002/advs.202416963 (PMC12079500; doi:10.1002/advs.202416963)
Supplement: Supplementary file 1 — Supporting Information [file ADVS-12-2416963-s001.docx]

**Intracellular Peptide Library Screening for Irreversible Covalent Transcription Factor Inhibitors**

Andrew Brennan^1^, Scott Lovell^1^, Keith Vance^1^ and Jody M Mason^1^

^1^Department of Life Sciences, University of Bath, Bath BA2 7AY, United Kingdom

Address correspondence to JMM (j.mason@bath.ac.uk)

**Experimental Section**

**AlphaFold predictions:** The Google Colab platform, v.2.1 was used to produce the AlphaFold-multimer prediction of the cJun/HW1 structure. No template information was used. 5 models were produced with a high degree of structural similarity, indicated by a range of pairwise RMSD values between 0.27 and 0.375 Å. pTM score: 0.68; ipTM score: 0.72. AlphaFold Server was used to produce the AlphaFold3 prediction of the cJun/HW31 structure. 5 models were produced with a high degree of structural similarity, indicated by a range of pairwise RMSD values between 0.243 and 0.569 Å. pTM score: 0.75; ipTM score: 0.77.

**Library Construction and TBS Assay**: Library inserts were produced using PCR fill-in reactions from synthesised primers (Merck) with degenerate codons at the desired positions to produce the correct residue options. The library was subcloned using NheI and AscI sites into the pET24a plasmid. The primers used were Forward: 5’- ATGGCTAGCCTGGAACAGCGCGCGGAAGAACTGGCGCGCGAAAACGAAGAATKKKRTYGTKRTKSCKRTKRTTKKKKCGTGGAAGAAGATGTGCTGGAAGAAG

-3’ and Reverse 5’- TGAGGCGCGCCCAGTTTCTCCAGCTGTTTCTGCAGATCTTCAATTTCTTTGCGCAGCGCATAGTTGCGTTCTTCCAGCTGTTCAATTTCTTCTTCCAGCACATC

-3’. The PCR-fill in reaction therefore generates a library encoded with the following sequence: 5’- ATGGCTAGCCTGGAACAGCGCGCGGAAGAACTGGCGCGCGAAAACGAAGAATKKKRTYGTKRTKSCKRTKRTTKKKKCGTGGAAGAAGATGTGCTGGAAGAAGAAATTGAACAGCTGGAAGAACGCAACTATGCGCTGCGCAAAGAAATTGAAGATCTGCAGAAACAGCTGGAGAAACTGGGCGCGCCTCA-3’.

Positions **d3** and **d4** were randomised using a TKK codon (T G/T G/T) to encode Phe, Cys, Leu or Trp. Positions **e3**, **g3**, **b3** and **c3** were randomised using a KRT codon (G/T A/G T) to encode Asp, Gly, Tyr or Cys. Position **f3** was randomised using a YGT codon (T/C G T) to encode Arg or Cys. Position **a3** was randomised using a KSC codon (G/T G/C C) to encode Ala, Ser, Cys or Gly. Position **e4** was randomised using a KKC codon (G/T G/T C) to encode Phe, Val, Cys or Gly.

The library DNA was transformed into NEB 10-beta electrocompetent *E. coli* cells. The following equation was utilised to determine library coverage by the number of single colonies: $E=100\times{(1-\frac{1}{n})}^{m}$ where E is the percentage of the library missing, m is the number of colonies collected and n is the library size. This showed that from 2979580 library colonies collected there was a 99.9% probability that the full library was present. Library DNA quality was assessed by sequencing both the DNA pool and a number of single colonies to show degenerate codons in the correct positions in the pool and to show a diversity of library members from single colonies. For library screening, “TBS cells” were produced containing the TRE-mDHFR (pES300d) and cJun bZIP (pES230d) plasmids; these cannot grow on selective media as the essential transcription of the TRE-mDHFR is blocked by cJun TRE binding within the coding region. Next, the antagonist library plasmid pool was transformed into TBS cells such that by plating out under selective conditions, cells only grew if they expressed a plasmid encoding an antagonist capable of preventing a cJun-TRE interaction. Dilutions of the recovery media were also plated on non-selective M9 agar to determine the transformation efficiency and confirm full library coverage. During these experiments, 312 colonies formed from SHuffle Express cells, and 127 colonies from BL21-Gold cells on selective agar. These were separately collected and transferred to selective liquid media and passaged for continuous growth and therefore competition selection. During this process, cells expressing the most effective antagonists impart a growth advantage; these dominate the bacterial pool over time, which was monitored by DNA sequencing.

Selective pressure was applied by growing the bacteria in M9 minimal media with TMP (2-8 µM) alongside ampicillin, kanamycin and chloramphenicol to maintain the required plasmids, and IPTG (1 mM) to induce protein expression. At each passage step, a sample of the culture was plated on LB agar (supplemented with kanamycin to maintain the antagonist pET24a plasmid) to select and sequence individual colonies, and a DNA pool was also sequenced. This allowed the occurrence of library members to be monitored throughout the assay.

**Peptide synthesis**: Peptides were synthesised using a Liberty Blue microwave peptide synthesiser (CEM) at a 0.1 mmol scale on ChemMatrix Rink amide resin using standard Fmoc solid-phase methodology. Coupling was performed using 5x amino acid, 4.5x PyBOP and 10x diisopropylethylamine in dimethylformamide (DMF, 5 mL). Deprotection was performed using 20% piperidine in DMF. Peptides were acetylated at the N-terminus by a final reaction with 3x acetic anhydride, 4.5x diisopropylethylamine in DMF for one hour at room temperature. FAM was added to the N-terminus by double coupling with 5x 5(6)-carboxyfluorescein (Merck), 5x diisopropylethylamine and 5x PyBOP for a total time across both couplings of 24 hours at 50°C. For lactamised peptides, the relevant K and D positions were orthogonally protected using Lys(Mtt) and Asp(O-2-PhiPr). The sidechains of these residues were selectively deprotected by washing the resin with dichloromethane (DCM) x3, 2% trifluoroacetic acid (TFA) in DCM x10, DCM x3 then DMF x3. The deprotected sidechains were coupled in 4.5x PyBOP and 10x diisopropylethylamine in DMF for 5 hours at 50 °C. The resin was dried, and the same reagents were added for a second reaction for 16 hours at 50 °C. Incubation in a cleavage mixture (95% TFA, 2.5% triisopropylsilane, 2.5% H_2_O, 10 mL) for 4 h at room temperature cleaved the peptide from the resin and removed side chain protecting groups. The resin was removed by filtration and cleaved peptides were precipitated in diethyl ether at -80°C and centrifuged. This pellet was washed a further four times with diethyl ether before it was dried overnight at room temperature. Peptides were resuspended in 1:1 water:acetonitrile (0.1% TFA) before purification using RP-HPLC with a Jupiter Proteo column (4-μm particle size, 90 Å pore size, 250 × 10 mm; Phenomenex) using a water:acetonitrile gradient (0.1% TFA). Peptide masses and purity (>90%) were verified by electrospray ionisation mass spectrometry. Upon solubilising in buffer, peptides were centrifuged to ensure insoluble/aggregated material was removed before peptide concentration determination.

**HW32 synthesis:** HW31 (8 mg) was resuspended in 16.8 mL 100 mM potassium phosphate buffer, pH 8 in a 15 mL conical tube. 1.2 mL of a 15 mM solution of Ellman’s reagent prepared in the same buffer was added to a final concentration of 100 µM protein and 1 mM Ellman’s. The reaction was shaken at 100 rpm for 30 minutes at 25 °C before purification of the peptide-Ellman’s adduct using RP-HPLC (as above). The purified peptide-Ellman’s adduct (7.2 mg) was resuspended in 15.5 mL 100 mM sodium phosphate buffer, pH 8 in a 15 mL conical tube to give a 100 µM solution. 0.3 g of Na_2_CO_3_ was added and vortexed to dissolve. 1408 µL of HMPT was added and the reaction was shaken at 100 rpm for 1 hour at 25 °C. Reactions were monitored with LC-MS and the resulting dehydroalanine containing product was purified by RP-HPLC (as above).

**HW33 bis-alkylation:** Crude cleaved peptide was resuspended in 100 mM NH_4_HCO_3_ pH 8 at 4 mg/mL. 1.5 equivalents of TCEP were added before incubation at 40°C for one hour. 1.2 equivalents of mDBMB were added from a 0.1 M stock in acetonitrile and the reaction was shaken at 100 rpm for 2 hours at 40°C. TFA was then added to a final concentration of 0.01% to facilitate methionine alkylation at low pH and the reaction was shaken at 100 rpm for 2 hours at 40°C. Reactions were monitored with LC-MS and the resulting bis-alkylated product was purified by RP-HPLC (as above).

**Circular dichroism (CD)**: An Applied Photophysics Chirascan was used for CD measurements, with a 200 µL sample in a 1 mm path length CD cell. Protein/DNA samples were suspended in 20 mM potassium phosphate,150 mM potassium fluoride at pH 7.4 with or without 5 mM TCEP. cJun was added to TRE DNA (5’-GTCAGTCAGTGACTCAATCGGTCA, Merck) before addition of antagonist peptide and these samples were equilibrated for 30 minutes before measurement.^[13]^ For full spectra, three scans between 190 and 260 nm (265-320 nm for DNA binding experiments) were collected with a bandwidth of 1 nm and data sampled at a rate of 0.5 s^-1^. These scans were averaged and converted to molar residue ellipticities (MRE). Thermal denaturation experiments were performed by measuring the ellipticity at 222 nm over a 1 to 95°C gradient at 1°C increments. Post-melt scans at 20°C confirmed the transitions were reversible as they overlaid within 5% of the pre-melt scan. The resulting thermal denaturation curves were converted to MRE and fitted to a two-state model, derived via modification of the Gibbs–Helmholtz equation to determine the melting temperature (*T_m_*) ^[26]^.

**Size exclusion chromatography:** Peptide samples were prepared at 20 µM total peptide concentration in 20 mM potassium phosphate, 150 mM sodium chloride (pH 7.4) buffer. 100 µL samples were injected onto a Superdex Peptide 10/300 GL column (GE Healthcare Life Sciences) at a 0.5 mL min^-1^ flow rate and elution profiles were recorded by monitoring absorbance at 215 nm.

**Isothermal titration calorimetry:** Binding thermodynamics were measured using a Microcal PEAQ-ITC (Malvern Instruments) in a buffer consisting of 20 mM potassium phosphate, 150 mM sodium chloride and 2 mM TCEP (pH 6.5). Sequential 2 µL injections of HW33 (20 µM) were added to cJun (2 µM) in the cell at 25°C. Data from a control experiment whereby HW33 (20 µM) was injected into buffer in the cell were collected and subtracted. The heat change data were fit using Microcal Analysis software to a one site binding model. Thermodynamic parameters are given as an average of three independent experiments and errors are given as one standard deviation.

**Fluorescence polarisation:** Fluorescence polarisation was measured in a CLARIOstar fluorescence microplate reader (BMG Labtech), using black polystyrene, non-binding surface 96-well half area plates (Corning) in a 20 mM potassium phosphate, 50 mM sodium chloride, 5 mM MgCl_2_, 5 mM TCEP, pH 7.4 buffer. FAM-TRE DNA oligos were purchased from Merck with the sequences: FAM-TRE.Forward: 5’[6FAM]-TCTCTGACTCAGAGA; FAM-TRE.Reverse: 5’[6FAM]-TCTCTGAGTCAGAGA. 10 µM of each oligo was prepared in water and then annealed by heating to 95°C for 10 minutes before cooling slowly to room temperature to give a 10 µM duplex stock. Samples containing 10 nM FAM-TRE and a serial dilution of cJun (3000-4.9 nM) were prepared and incubated at room temperature for 1 hour before transferring to the assay plate for measurement. Data were fitted to a one site binding model. For antagonism experiments, the cJun concentration required for a 70% response was selected. Samples containing 10 nM FAM-TRE, 625 nM cJun and a serial dilution of HW1 or HW31 (10000-20 nM) were prepared and immediately transferred to the assay plate for time-course FP measurement. All measurements were carried out in quadruplicate and reported as the mean with errors given as one standard deviation.

**Covalent conjugation assays:** Initial tests were performed by incubation of HW32 and HW33 (40 µM) with cJun bZIP (20 µM) in 50 mM potassium phosphate butter, 150 mM NaCl, pH 7.4 at 37°C, shaking at 450 rpm. Aliquots were taken over time and quenched by addition of SDS-PAGE loading buffer and heating to 95°C for 10 minutes before running the samples on a 12% acrylamide gel. This reaction was repeated for the addition of HW33 (40 µM) into CREB1 (20 µM). Further conjugation assays were performed across a range of concentrations of HW33 (10-100 µM) with cJun (20 µM). Aliquots of these reactions were taken over time and quenched by snap freezing in liquid nitrogen and lyophilisation, after which the dry powder was resuspended in 50:50 water:acetonitrile + 1% TFA. The samples were analysed by LC-MS on a Luna C18(2) column (5-μm particle size, 100 Å pore size, 150 × 4.6 mm; Phenomenex). The % occupancy was determined by the ratio of conjugated cJun over total cJun as determined by the area under the peaks in the chromatogram measured at a 215 nm wavelength, with their identity confirmed by ESI-MS. The cJun occupancy was plotted over time for each concentration and the data were fitted to a single exponential equation to give a *k_obs_* for each peptide concentration tested. The *k_obs_* values were plotted against concentration and the data were fitted to a straight line (y-intercept fixed at 0) with the gradient equal to *k_inact_*/*k_d_*. For cell lysate conjugation experiments, SK-MEL-28 cells were resuspended in 50 mM potassium phosphate butter, 150 mM NaCl, pH 7.4 at a density of 4x10^6^ cells/mL and lysed by sonication before addition of the indicated concentrations of FAM-HW33 and cJun and subsequent incubation at 37°C, shaking at 450 rpm for 4 hours before analysis by SDS-PAGE.

**Cell culture:** SK-MEL-28 cells were purchased from American Type Culture Collection. Cells were cultured in RPMI 1640 medium (Fisher) supplemented with 10% fetal bovine serum (Fisher) at 37°C in 5% CO_2_.

**Antibodies:** Anti-cJun, Rabbit mAb and anti-β-actin, Rabbit mAb were purchased from Cell Signalling Technologies and Goat Anti-Rabbit IgG, HRP-conjugate was purchased from Merck.

**Cell imaging:** Cells were seeded at 40,000 cells per well in a 96-well plate and incubated for 24 hours before treatment with vehicle (1% DMSO in water) or peptide for 6 hours. The media was removed and the cells were washed 3x with PBS. 1 mL of paraformaldehyde (80 mg/mL in PBS) was added and incubated at room temperature for 10 minutes. The paraformaldehyde was removed and fixed cells were washed with 3x PBS. 500 µL of DAPI (1 µg/mL) was then added and incubated at room temperature for 5 minutes following by a 3x PBS wash. Cells were imaged using an Evos FL digital inverted fluorescence microscope using brightfield and both GFP (for the FAM label) and DAPI fluorescence filters.

**Cell viability assay**: Cells were seeded at 15,000 cells per well in a 96-well plate and incubated for 24 hours before treatment with vehicle (1% DMSO in water) or peptide for 6 or 24 hours. The media was removed and the cells were washed twice with PBS before 50 µL of MTT (1 mg/mL in PBS, Merck) was added and incubated at 37°C for 30 minutes. The MTT solution was removed and 100 µL isopropanol was added to each well and rocked at room temperature for 5 minutes. Absorbance at 595 nm was measured using a Clariostar plate reader. Unless otherwise stated, all data are reported as an average of three independent biological experiments, with each data point determined in triplicate (i.e. 9 measurements) and reported as the mean with errors given as one standard deviation. For data with one independent measurement, each data point was determined in triplicate i.e. 3 measurements and reported as the mean with errors given as one standard deviation.

**Western Blot:** Cells were seeded at 60,000 cells per well in a 6-well plate and incubated for 24 hours before treatment with vehicle (1% DMSO in water) or peptide for 6 or 24 hours. Cells were trypsinised and collected from the well and washed with PBS. Cells were lysed using RIPA Lysis Buffer (Fisher) and the protein concentration was determined by Bradford assay (Bio-Rad). 20 µg of protein from each sample were loaded on a 12-20% SDS-PAGE gel and then transferred to polyvinylidene fluoride membrane (Merck). Bands were visualised using either anti-cJun or anti-β-actin antibodies as the primary at 1:1000 dilution, followed by anti-rabbit-HRP as the secondary at 1:5000 dilution and finally Immobilon Forte Western HRP substrate (Merck) was added and the chemiluminescence was detected using an Azure 400 imager.

**Statistical Analysis:** Unless indicated all data were fitted using OriginPro. Specific information relating to data presentation, sample size, and statistical method used to assess differences are provided within the methods or Figure legends as appropriate. No pre-processing of data (e.g., transformation, normalization, evaluation of outliers) was required.

**Supporting Information**

**
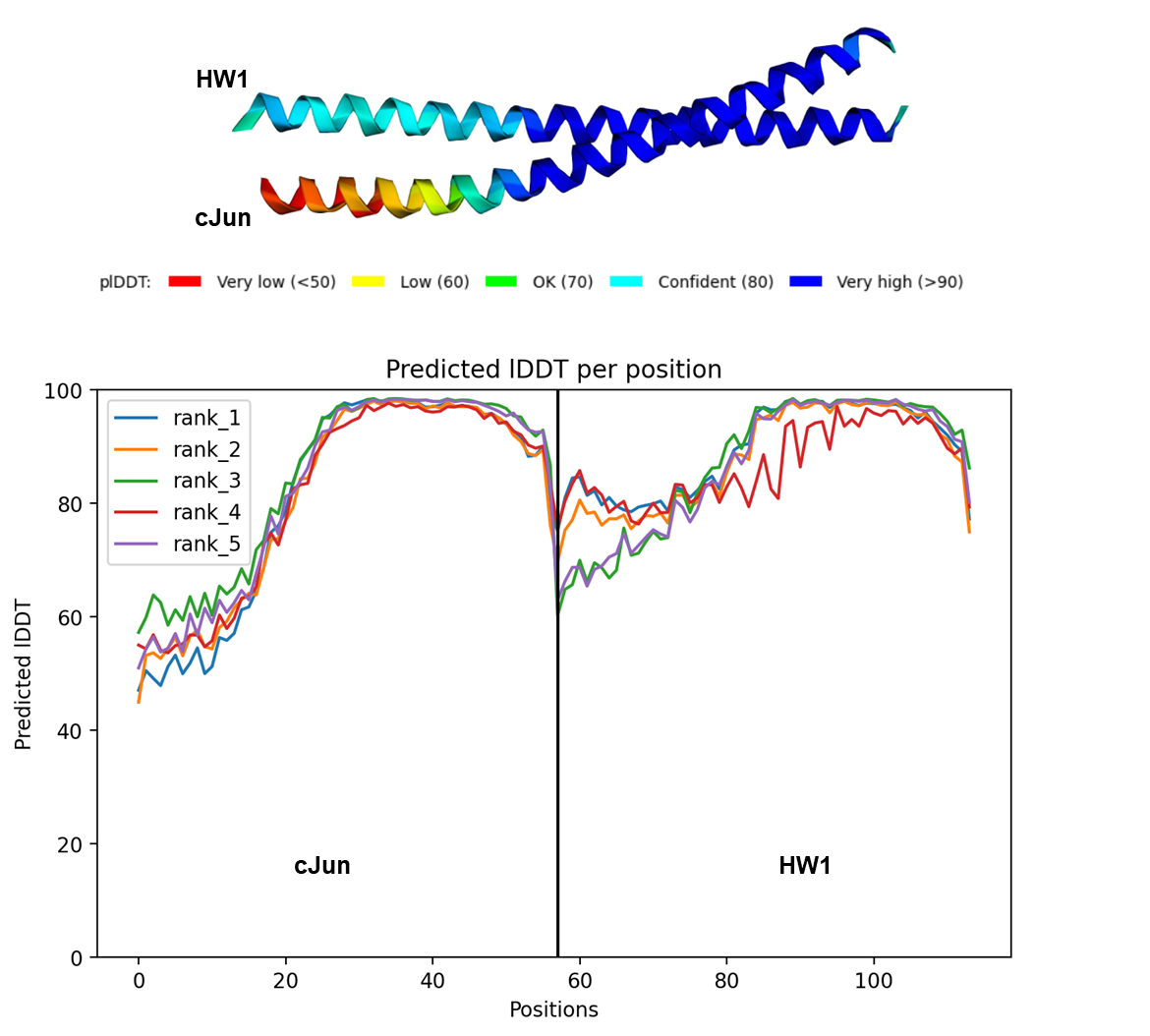
**

**Figure S1** – Output pLDDT values from the Alphafold-multimer (using the Google Colab platform, v.2.1.) prediction of the interaction between cJun and HW1.


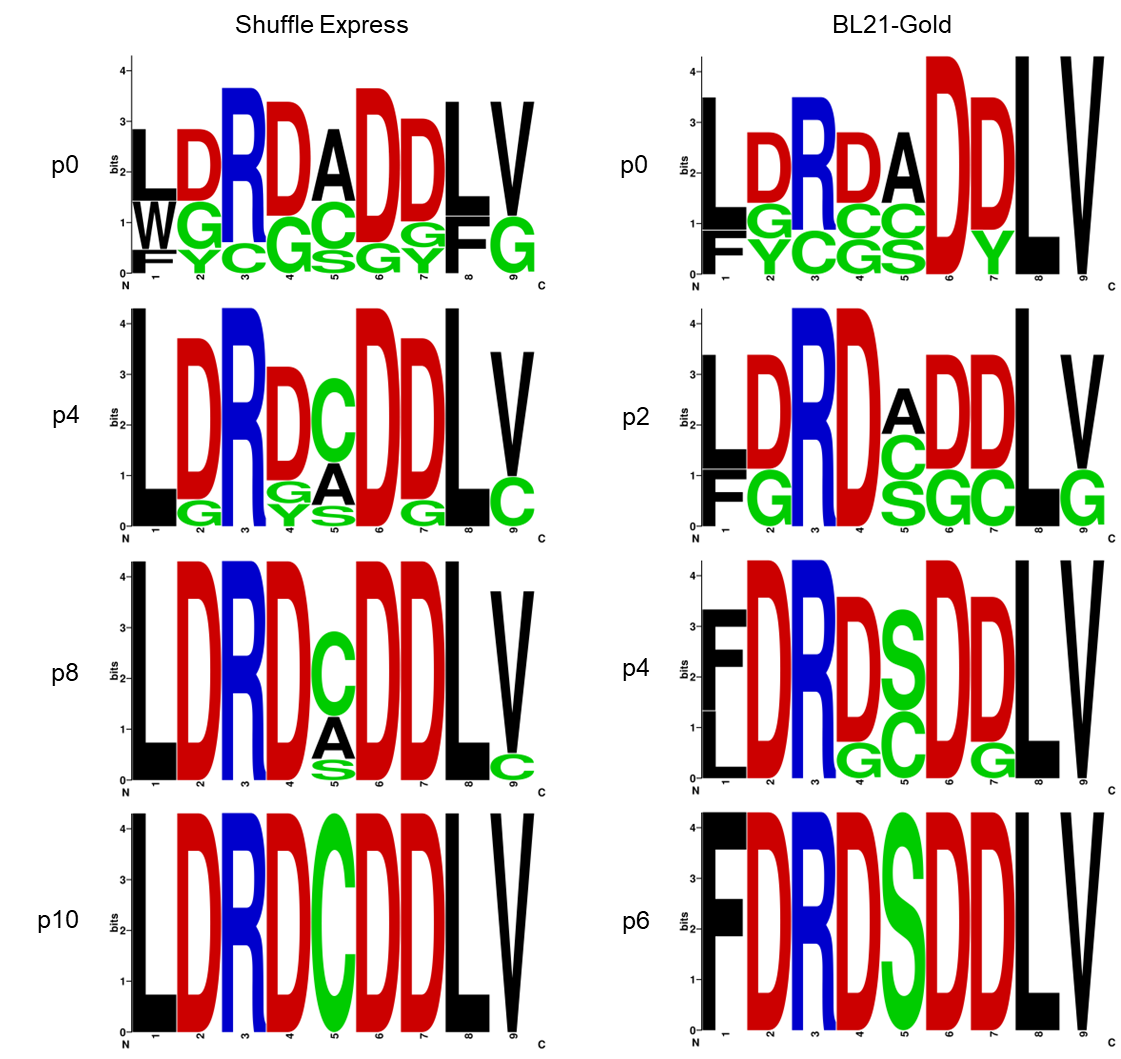


**Figure S2** – Sequence logos showing the relative abundances of the amino acid library options at various passage numbers during screening in Shuffle Express and BL21-Gold cells, determined from individual colony sequencing. Small sample sizes may not be fully representative of the library diversity but simply indicate sequences which were observed.

**
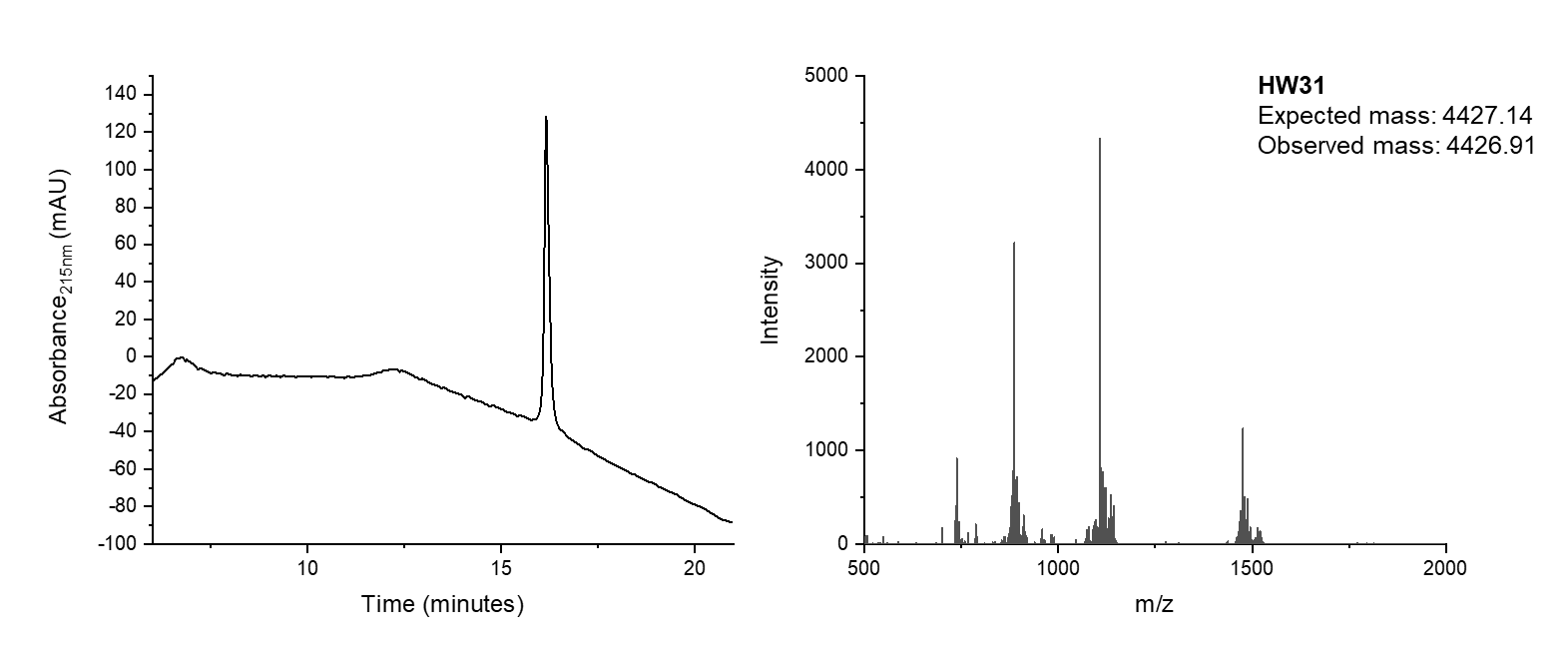
**

**Figure S3** – LC-MS analysis of HW31.

**
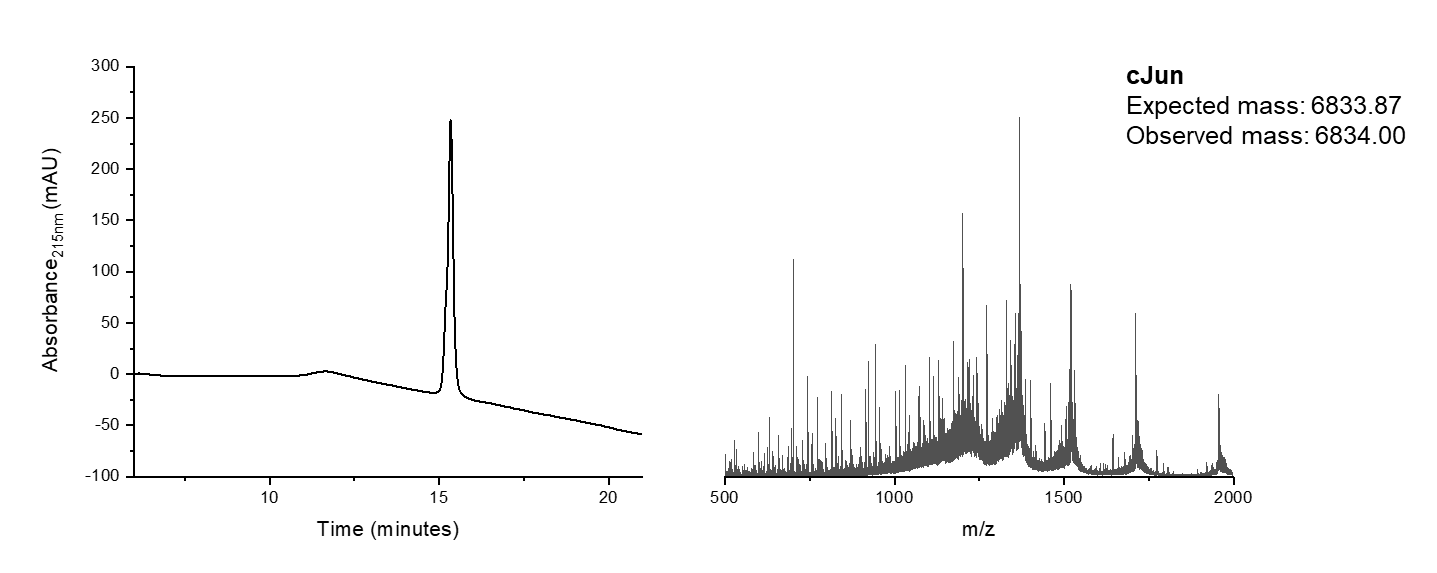
**


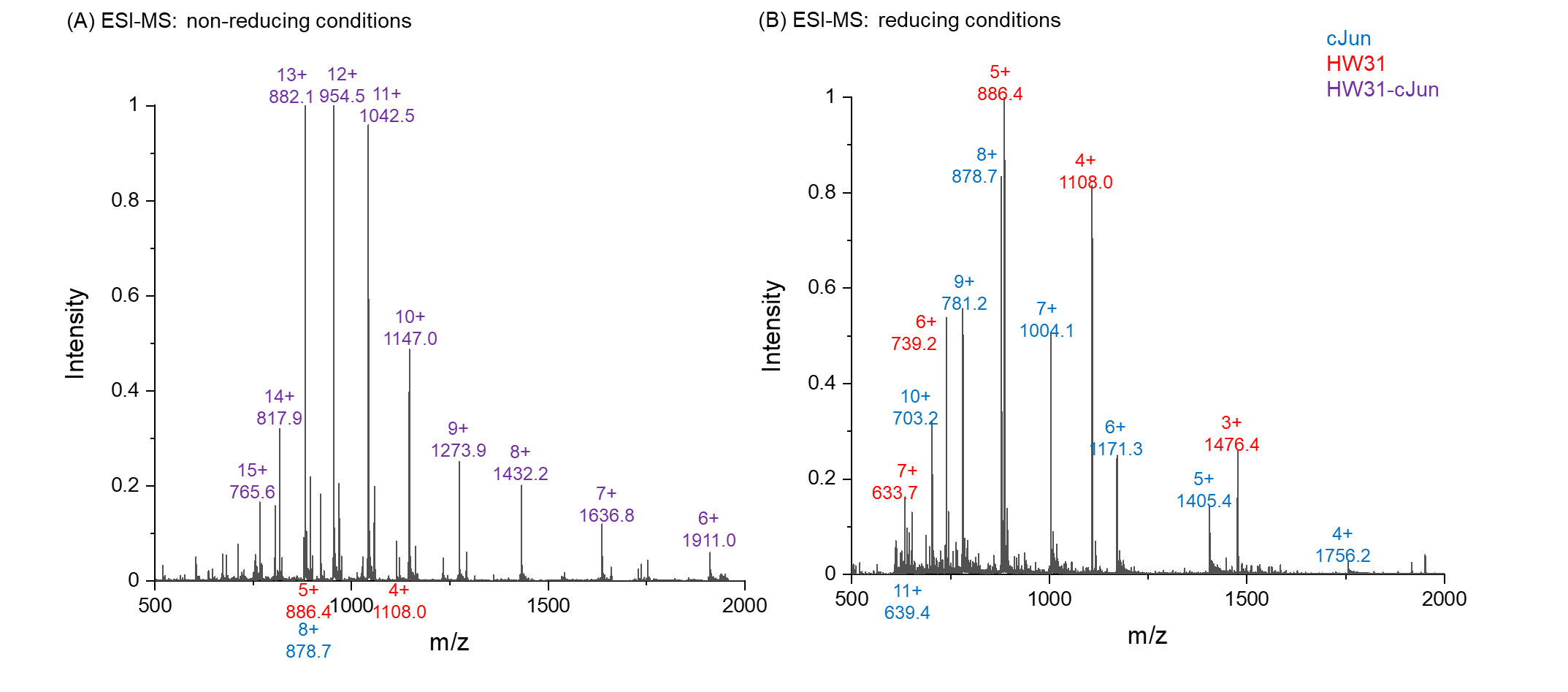
**Figure S4** – LC-MS analysis of the cJun bZIP domain

**Figure S5** - Mass spectra showing the mixture of HW31 (50 µM) and cJun (50 µM) in either (A) oxidising or (B) reducing conditions whereby the covalent attachment via a disulphide to form a single molecule or the two separate peptides are observed respectively.


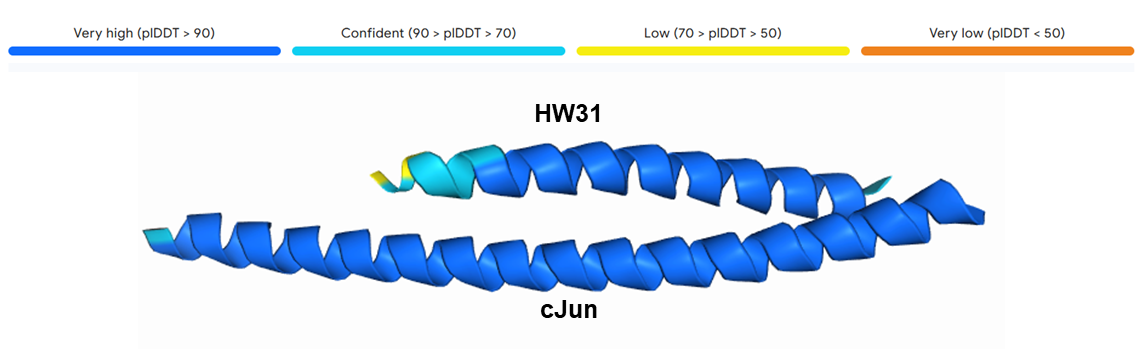


**Figure S6** – Output pLDDT values from the *Alphafold3 prediction of the interaction between cJun and HW31.*

**
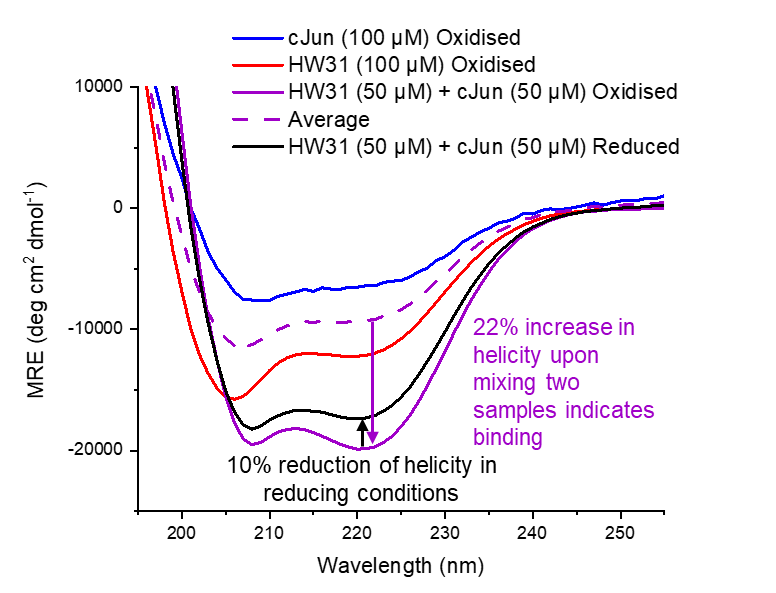
**


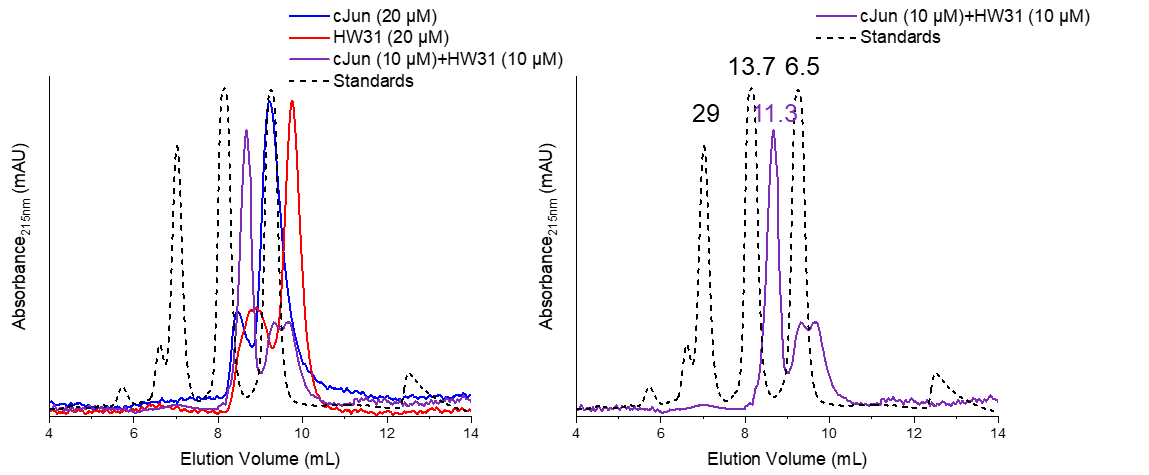
**Figure S7** – CD spectra of cJun and HW31 indicating an increase in helicity upon mixture of the two components which is decreased by 10% in reducing conditions which indicates the two peptides bind and adopt a coiled coil heterodimer structure with a disulphide bond formed between them which increases helicity. The “average” trace represents an averaged value of the two unbound components which is the predicted spectrum for no interaction.

**Figure S8** – Analytical size exclusion chromatography absorbance traces illustrate cJun/HW31 binding with a 1:1 stoichiometry. Both cJun and HW31 in isolation elute in two peaks, corresponding to a dominant monomer and a homodimer. Three peaks are observed for the cJun + HW31 mixture corresponding to a dominant heterodimer peak (eluting at 8.7 mL) and unbound monomers. No peaks were observed at earlier elution times indicating that no larger assemblies are formed upon binding. The standards used to validate the size of eluted peptides are aprotinin (6.5 kDa), ribonuclease A (13.7 kDa) and carbonic anhydrase (29 kDa); masses of eluted samples are indicated above the peaks in the right panel.

**Figure S9** – CD antagonism assay showing that the peak in the TRE DNA spectrum at 281 nm is shifted upon addition of cJun and that this shift is reversed in a dose-dependent manner upon addition of HW31, providing a readout of the amount of free/cJun-bound DNA.


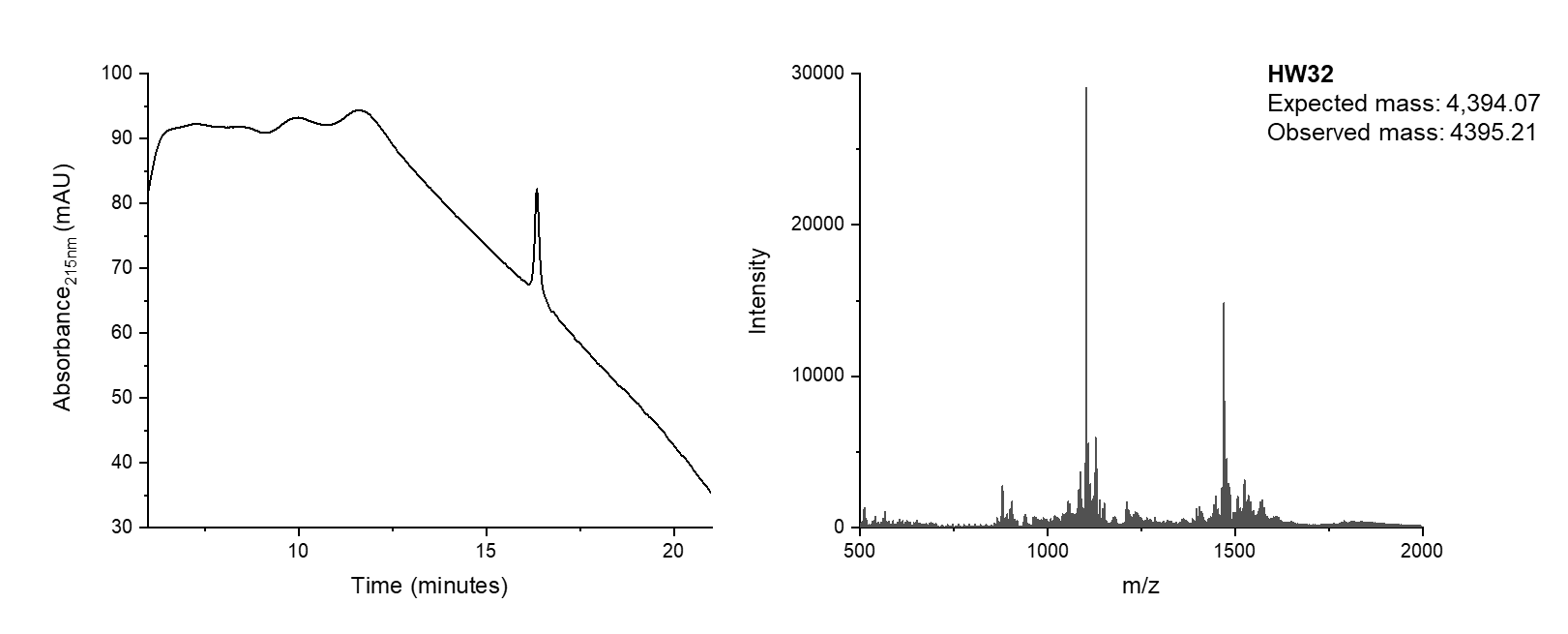


**Figure S10 ­**– LC-MS analysis of HW32.

**
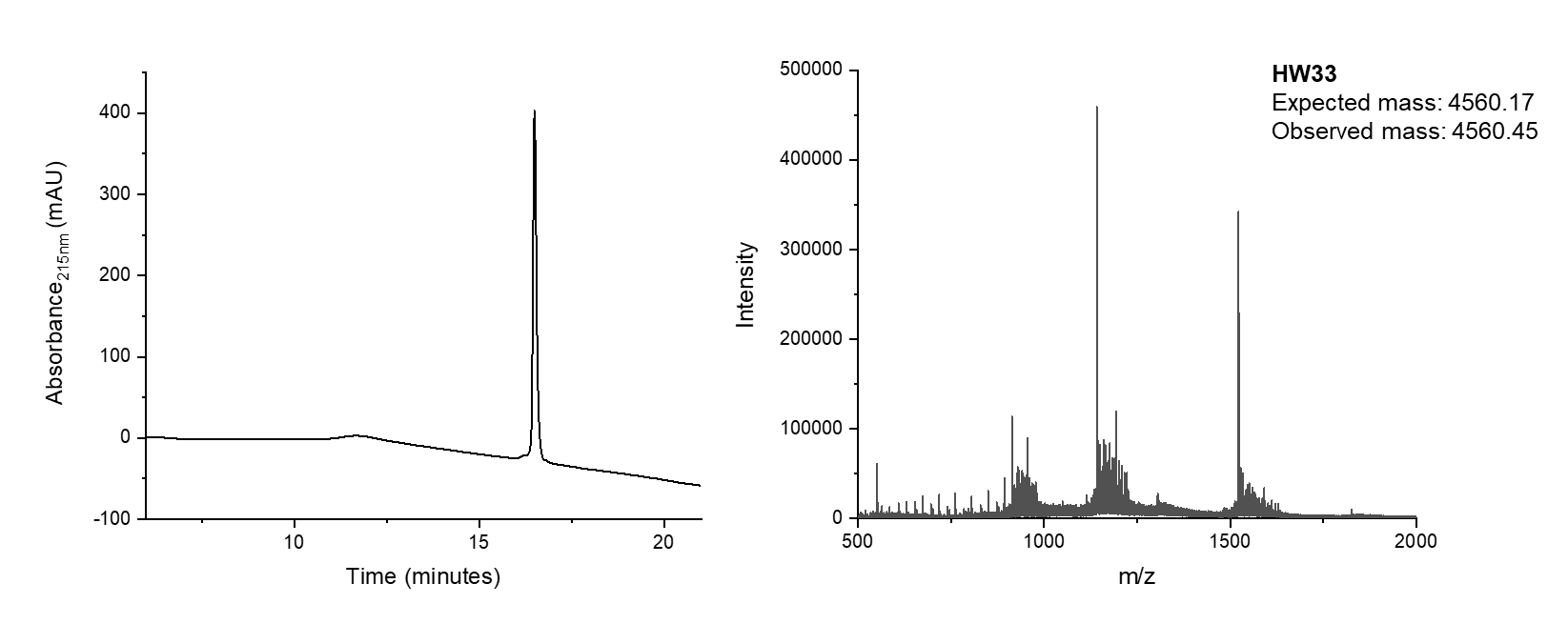
**

**Figure S11** – LC-MS analysis of HW33.


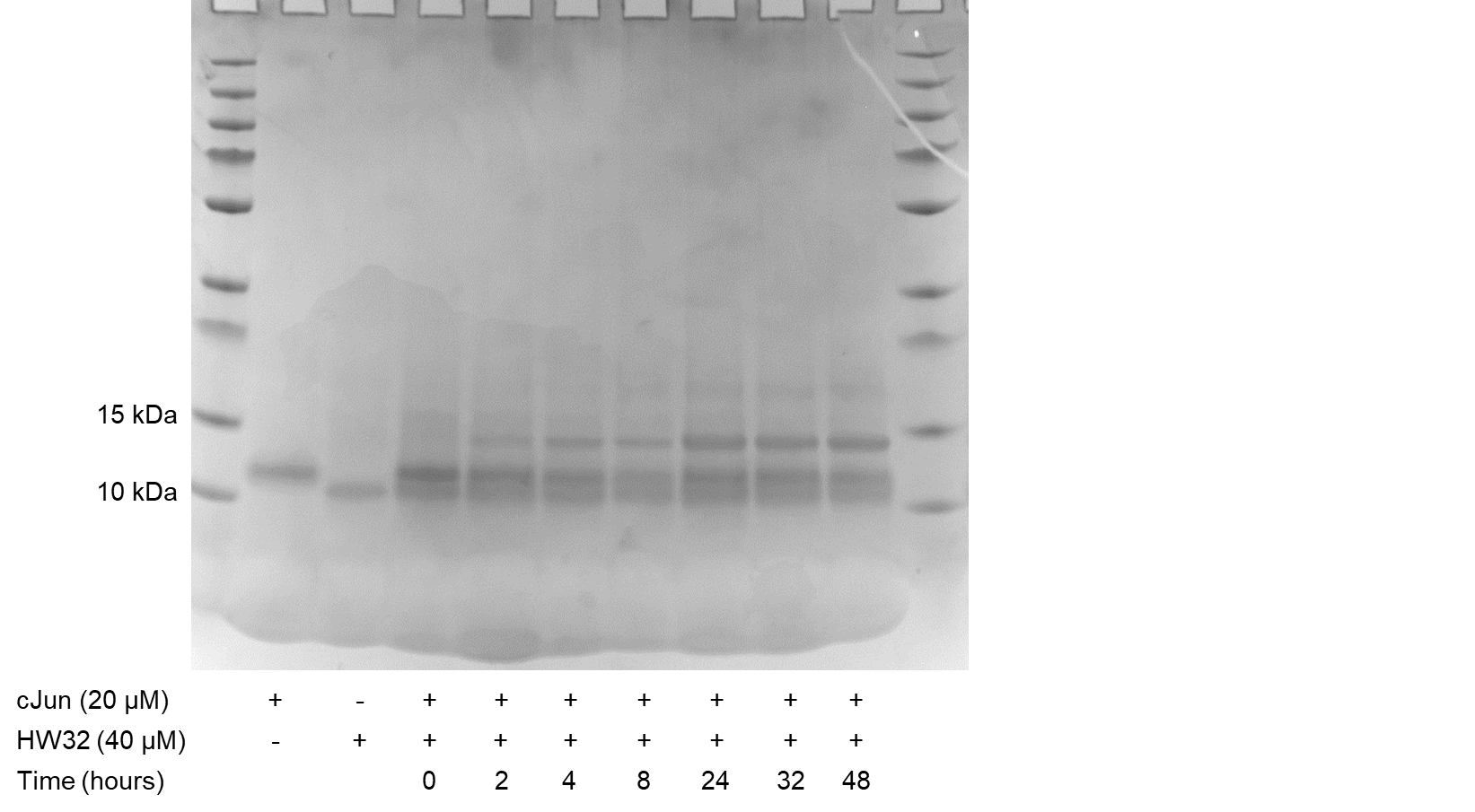


**Figure S12** - SDS-PAGE analysis of the reaction between cJun (20 µM) and HW32 (40 µM) over 48 hours which shows the formation of the covalently linked product. At later timepoints a higher molecular weight band was observed but this has not been further investigated due to our focus on HW33.

**
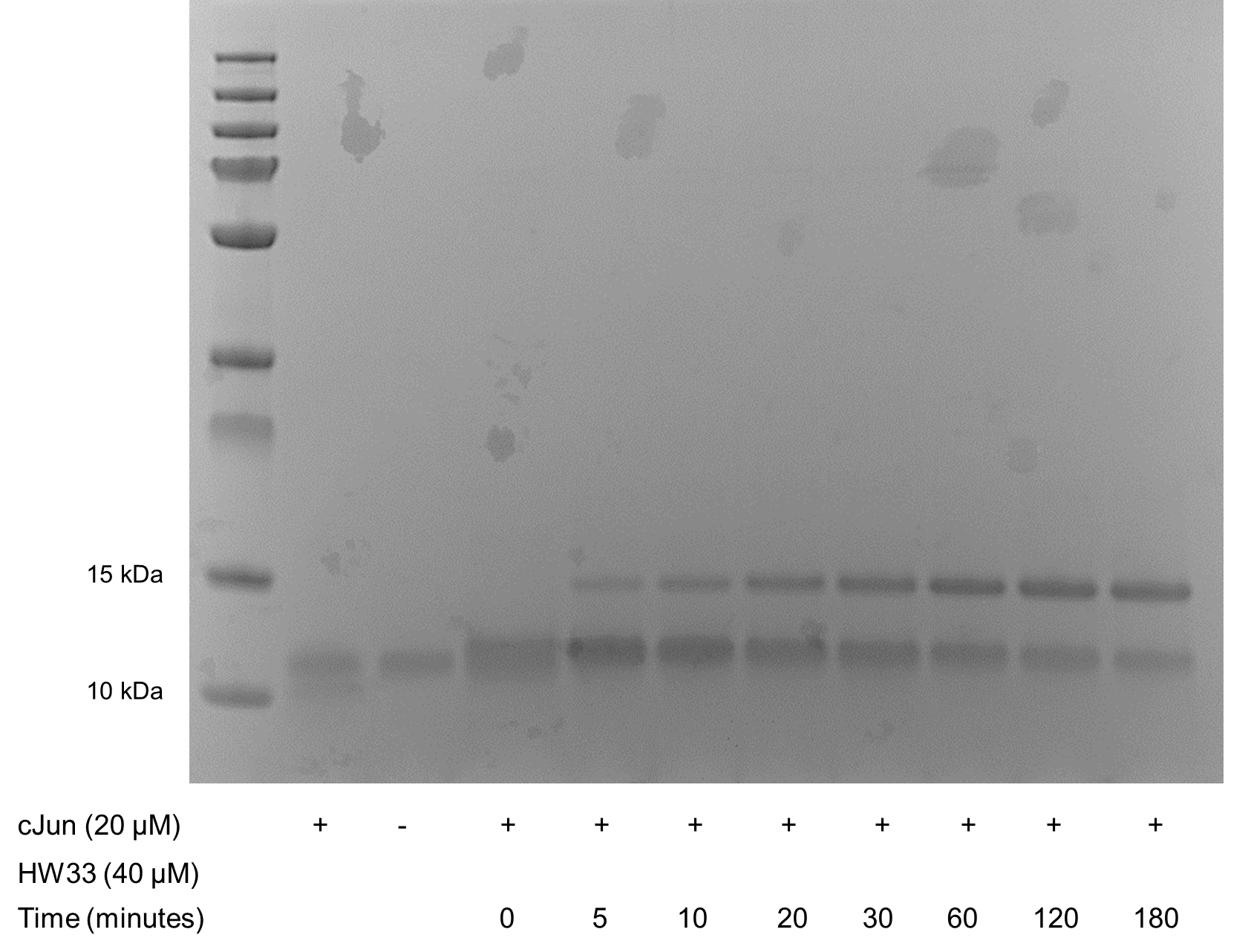
**

**Figure S13** - SDS-PAGE analysis of the reaction between cJun (20 µM) and HW33 (40 µM) over 180 minutes which shows the formation of the covalently linked product.

**Figure S14** - *k_inact_*/*k_d_* was determined by plotting the observed reaction rates against peptide concentration (R^2^ = 0.97). Linear fitting was undertaken using OriginPro.

**
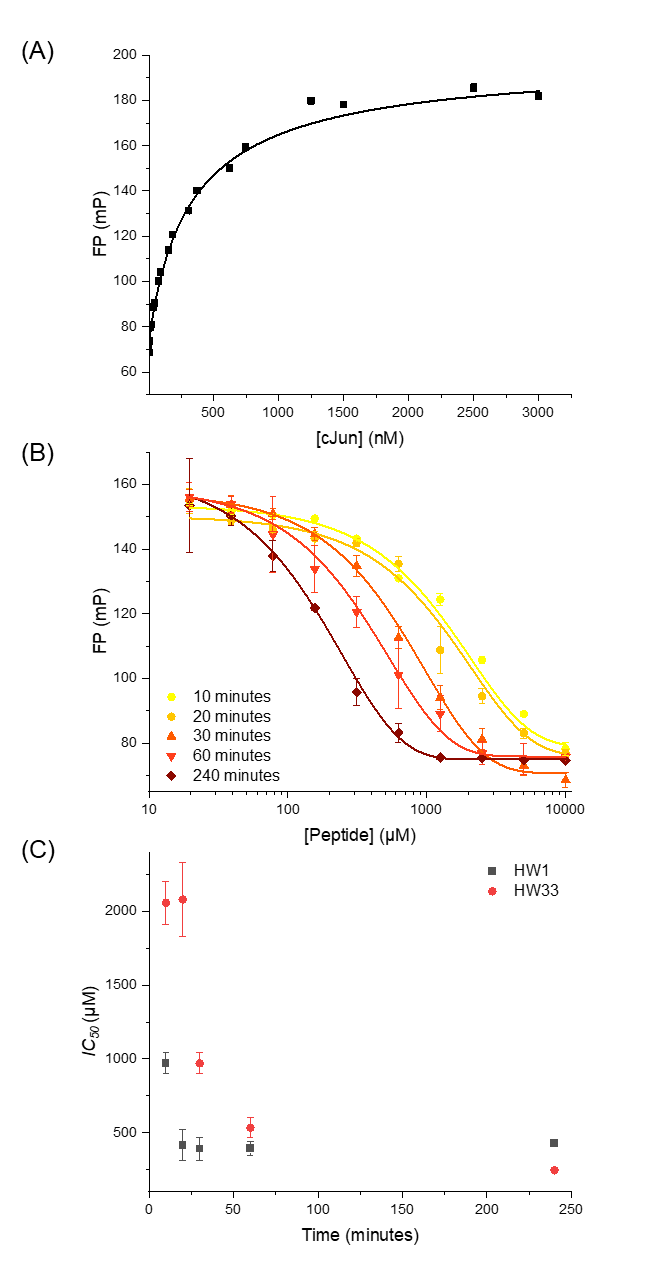
**

**Figure S15** – Fluorescence polarisation experiments show HW1 and HW33 antagonism of the cJun/TRE DNA interaction. (A) Fluorescence polarisation binding assay to illustrate cJun binding to FAM-TRE DNA with a *K_D_*=330±42 nM, which also identifies experimental conditions for antagonism experiments (data fitted to a Hill equation using OriginPro). (B) A time-course fluorescence polarisation experiment shows increasing efficacy of HW33 over time for the antagonism of cJun/FAM-TRE DNA (quadruplicate data fitted to a dose repose equation using OriginPro). (C) FP *IC_50_* values plotted over time for both HW1 and HW33 illustrate a time dependence on HW33 efficacy but not HW1, with HW33 showing improved antagonism over HW1 after 240 minutes.

**
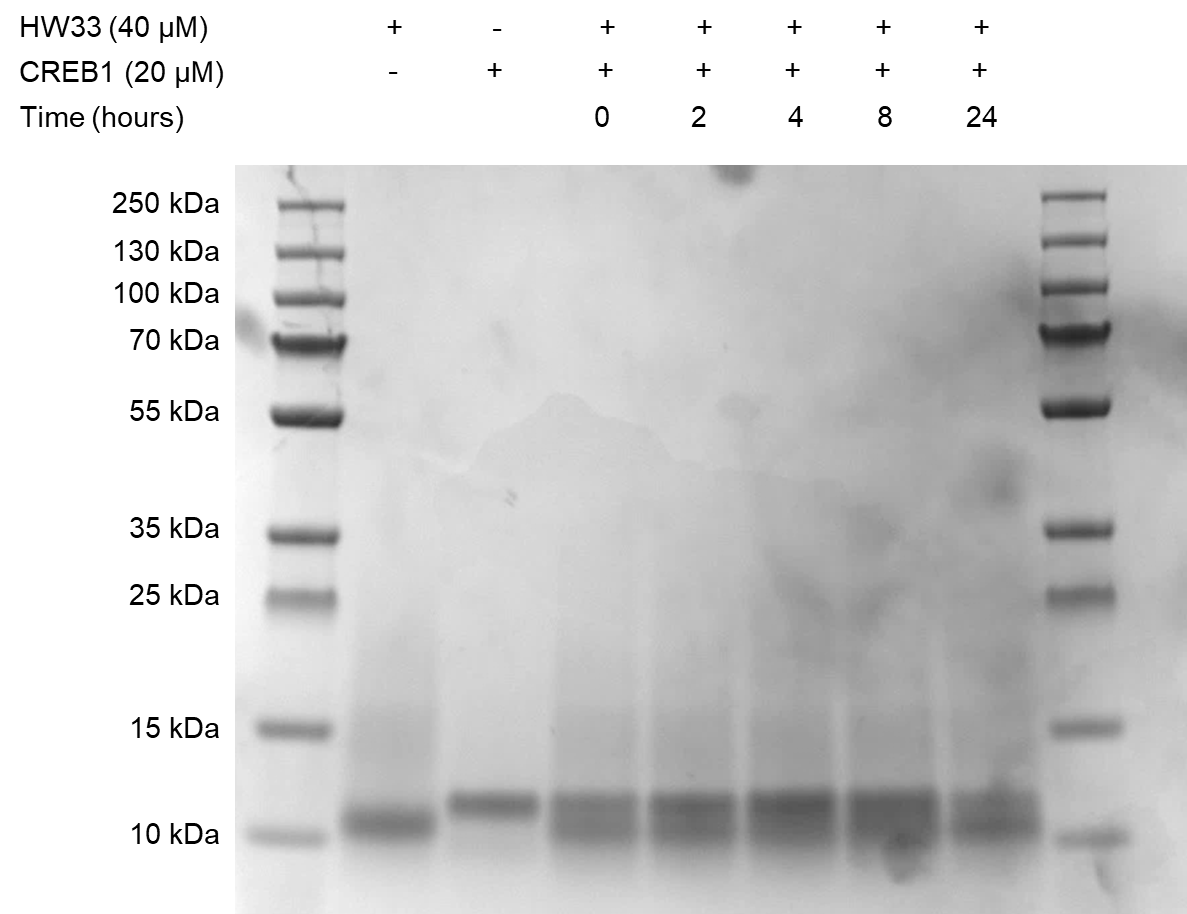
**

**Figure S16** - SDS-PAGE analysis of HW33 (40 µM) incubation with CREB1 (20 µM), an off-target bZIP, to show reaction specificity.


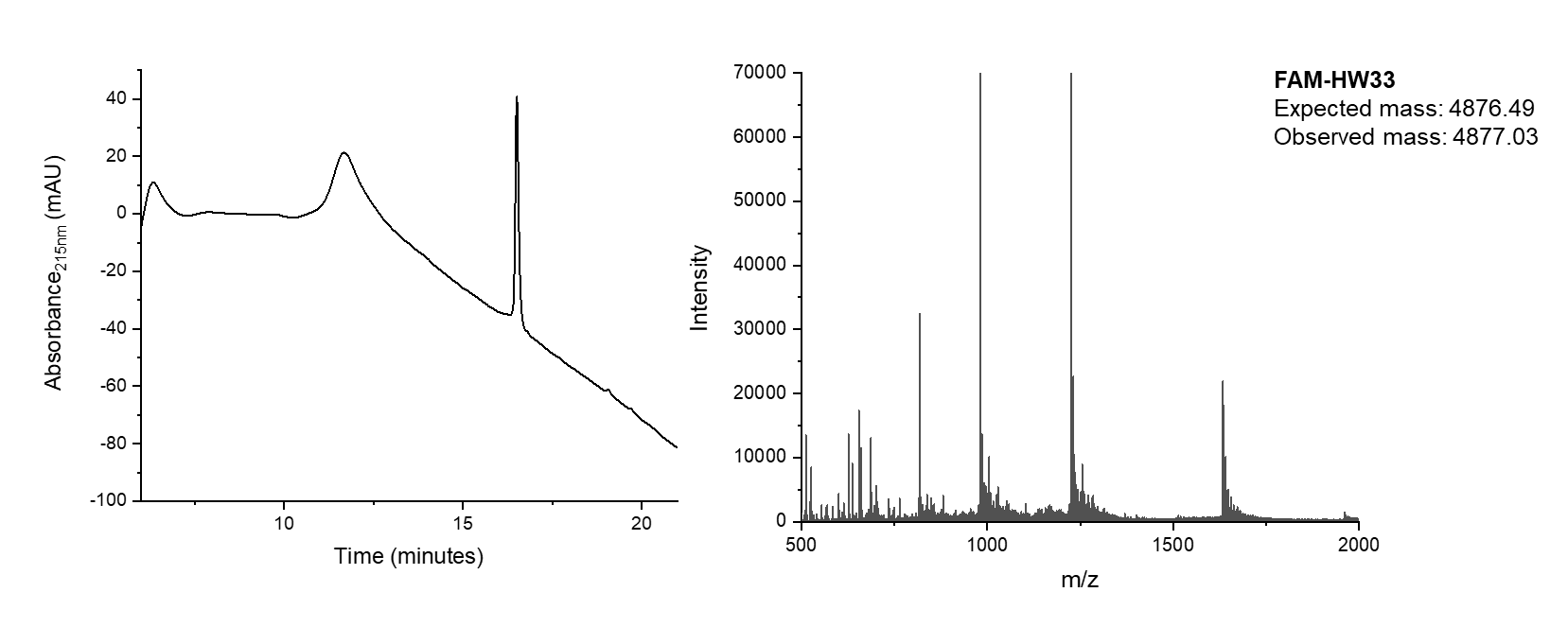


**Figure S17** – LC-MS analysis of FAM-HW33.


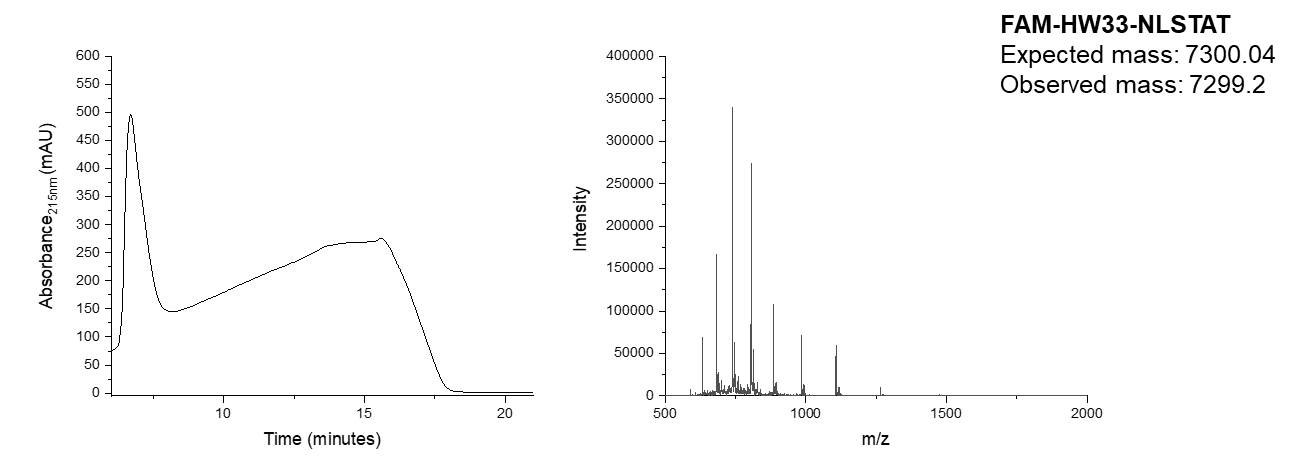


**Figure S18** – LC-MS analysis of FAM-HW33-NLSTAT


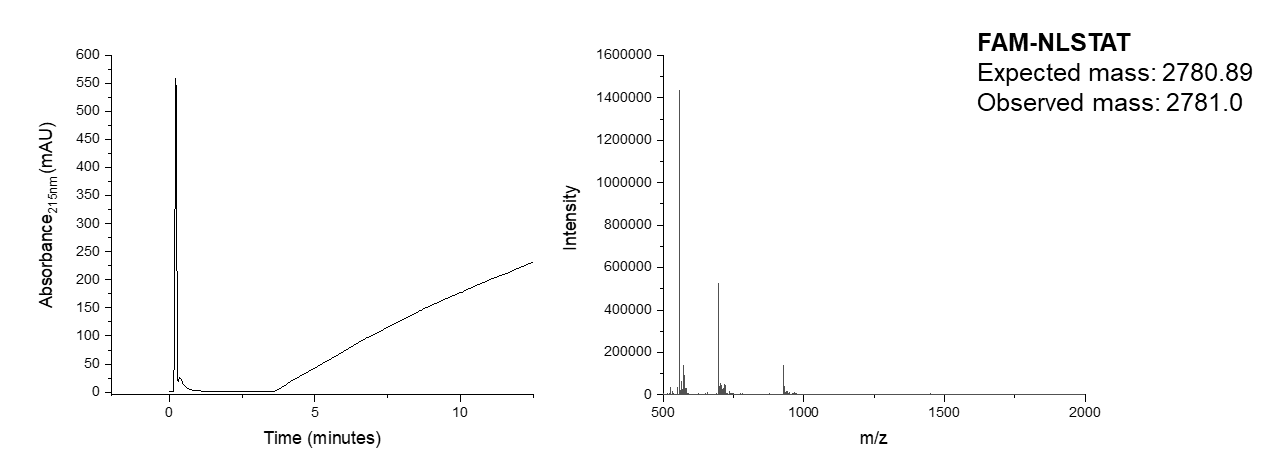


**Figure S19** – LC-MS analysis of FAM-NLSTAT


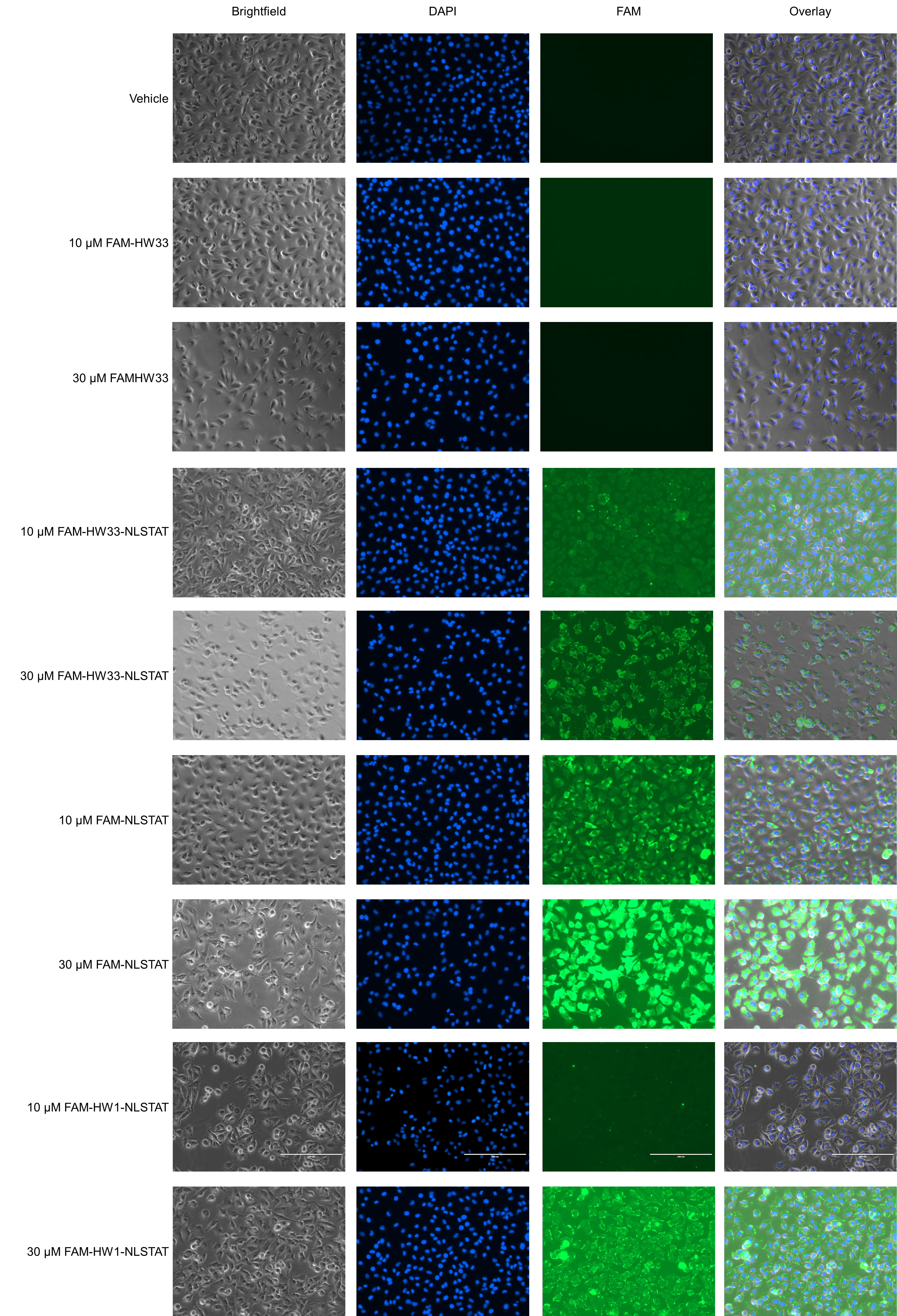


**Figure S20** - Imaging of SK-MEL-28 cells treated with indicated peptide concentrations for 6 hours at 37°C before fixing and DAPI counterstaining.

**Figure S21** - Viability of SK-MEL-28 cells after a 24 hour incubation with peptide across a range of concentrations. Data are normalised to vehicle control. FAM-HW33-NLSTAT data are the average of three independent experiments and FAM-HW33 data are the average from one independent experiment. Error bars are shown as one standard deviation.


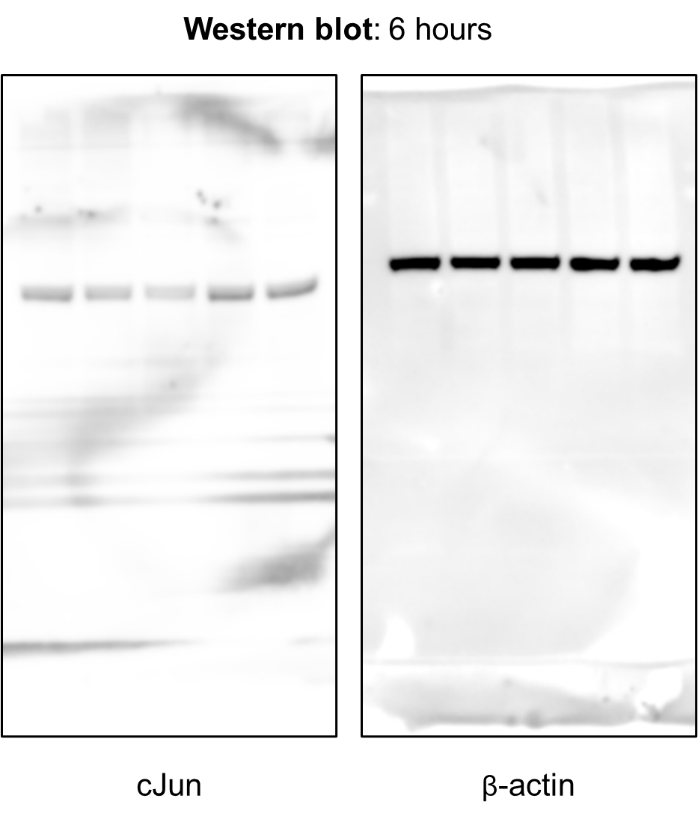


**Figure S22** – Uncropped images of western blot analysis of cJun and β-actin control levels in SK-MEL-28 cells treated by indicated peptide concentrations for 6 hours.


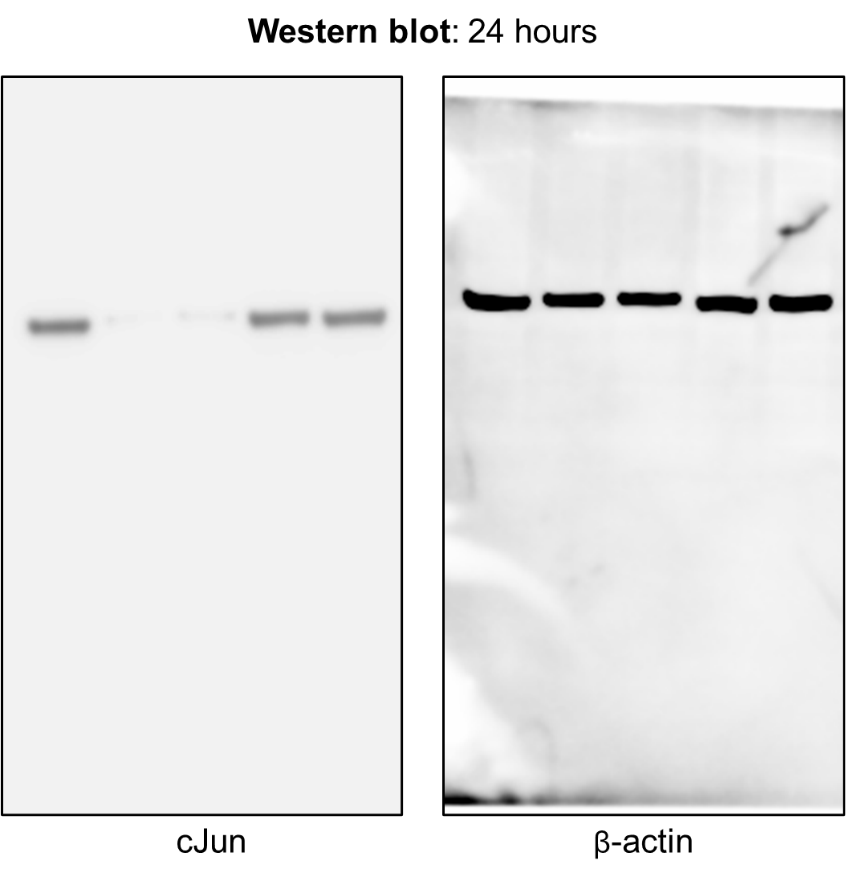


**Figure S23** – Uncropped images of western blot analysis of cJun and β-actin control levels in SK-MEL-28 cells treated by indicated peptide concentrations for 24 hours.


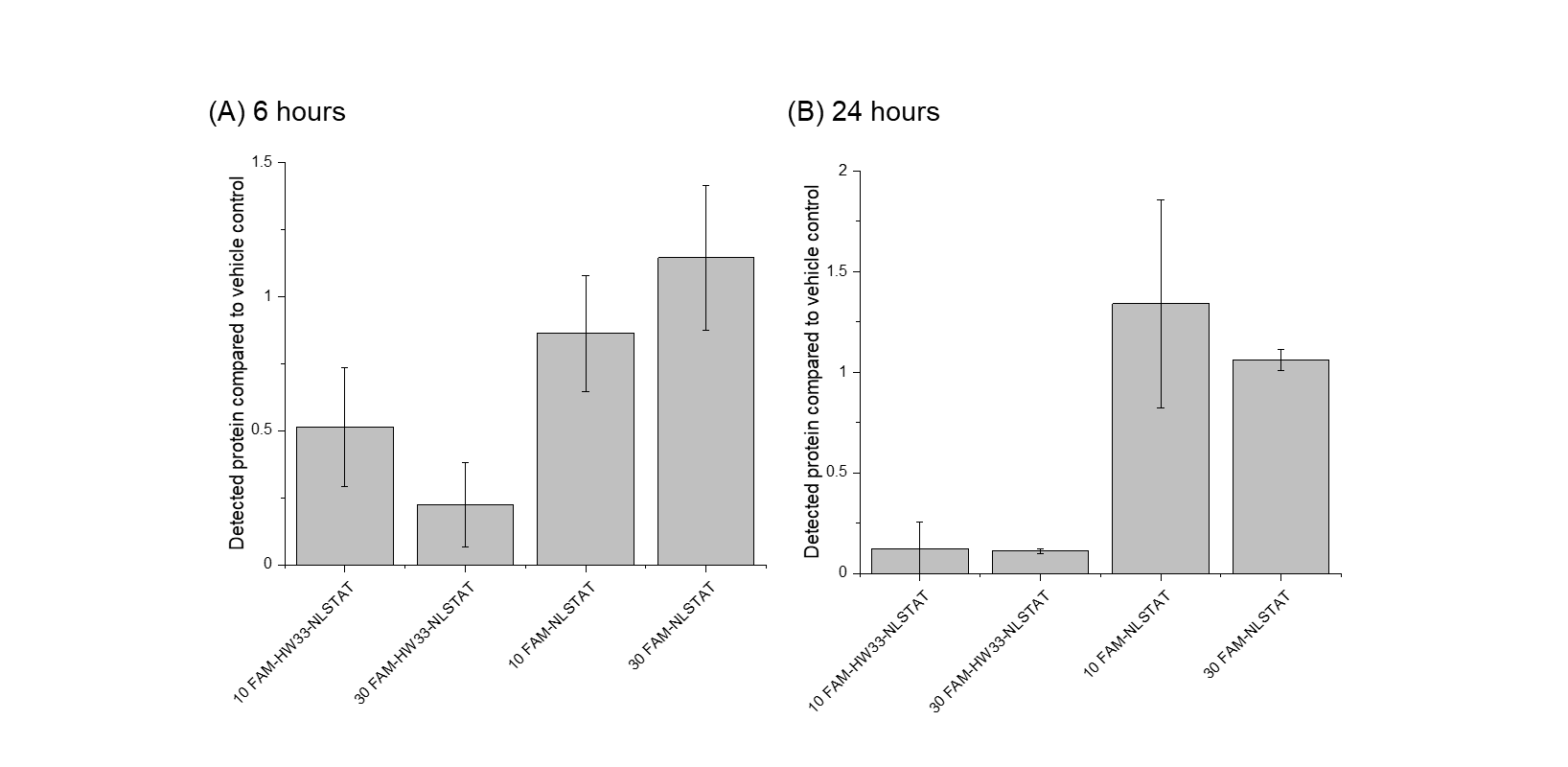


**Figure S24** – cJun protein levels determined by western blot at (A) 6 hours and (B) 24 hours of treatment with indicated peptide. Data are averages of three independent experiments using densitometry as determined using ImageJ and error bars are shown as one standard deviation.


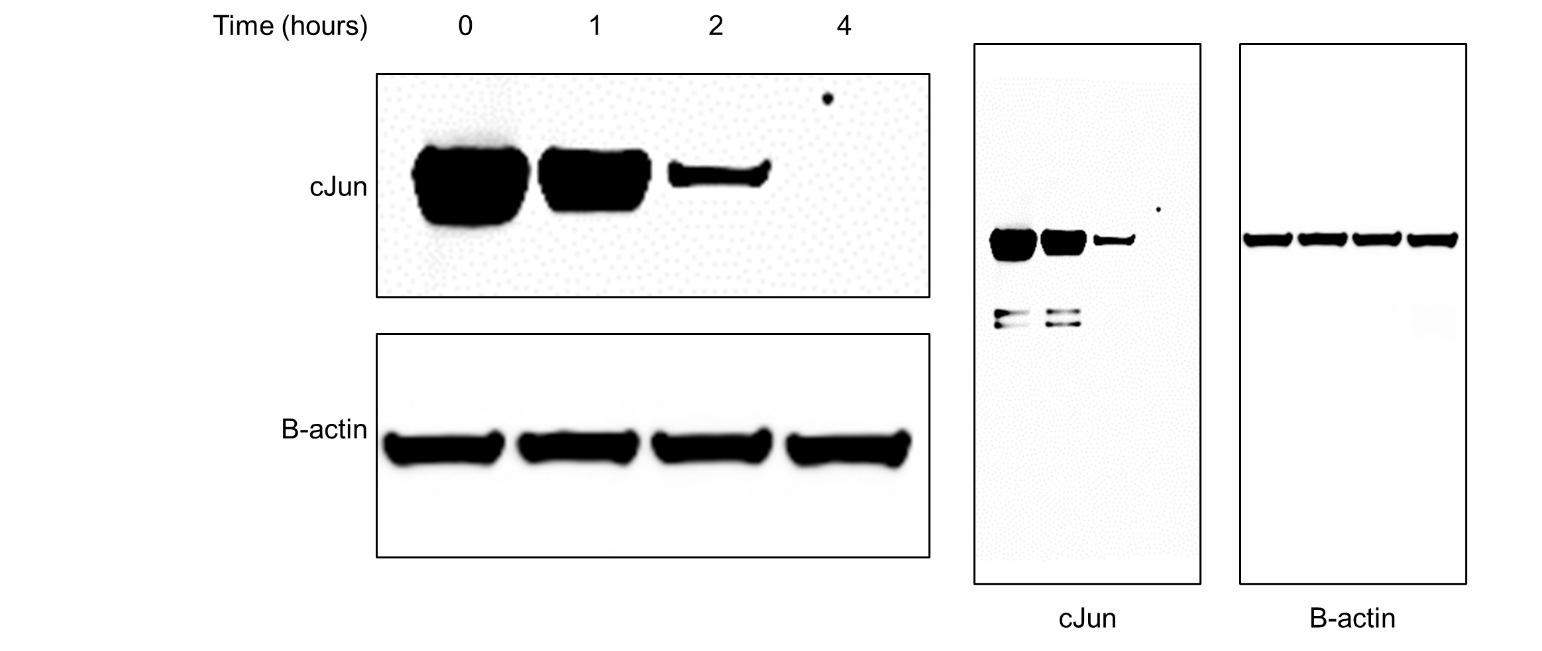


**Figure S25** – cJun protein levels in SK-MEL-28 cell lysate (with the addition of Roche cOmplete protease inhibitor cocktail) upon addition of 30 µM FAM-HW33-NLSTAT, determined over time by western blot. Cells were lysed by sonication in 20 mM KP, 150 mM NaCl, 5 mM TCEP, pH 7.4 and incubated at 37°C after peptide addition. Uncropped blots are also shown.

| **Antagonist** | **Antagonist-cJun heterodimer *T_m_* (°C)** | | | **cJun-TRE DNA**  ***IC_50_* (µM)** | | |
| --- | --- | --- | --- | --- | --- | --- |
|  | **oxidised** | **reduced** | **Δox-red** | **oxidised** | **reduced** | **Fold improved** |
| **HW1** | 70 | 73 | -3 | 11.8 ± 0.3 | 13.8 ±0.3 | 1 |
| **HW29** | 66 | 68 | -2 | 41.5 ± 2.0 | 40.1 ± 4.1 | 1 |
| **HW31** | 81 | 67 | 14 | 12.1 ± 0.3 | 48.3 ± 1.8 | 4 |

**Table S1 –** Parameters describing the ability of antagonists to inhibit the cJun-TRE DNA interaction in oxidising or reducing buffer. Errors given as one standard deviation.
